# Supplementary material for: Quantitative mobile gamma-ray spectrometry through Bayesian inference
Source: Nat Commun. 2026 Oct 26;17:10913. doi: 10.1038/s41467-026-78464-2 (PMC13710540; doi:10.1038/s41467-026-78464-2)
Supplement: Supplementary file 1 [file 41467_2026_78464_MOESM1_ESM.pdf]

Supplementary Information for

Quantitative mobile gamma-ray spectrometry through Bayesian inference

David Breitenmoser[1,2,*], Alberto Stabilini[1], Malgorzata Magdalena Kasprzak[1], Sabine Mayer[1]

[1]Department of Radiation Safety and Security, Paul Scherrer Institute (PSI), Forschungsstrasse 111, Villigen PSI, 5232, Switzerland

[2]Department of Nuclear Engineering & Radiological Sciences, University of Michigan, 2355 Bonisteel Blvd., Ann Arbor, MI 48109-2104, United States of America

[*]Lead and contact author: David Breitenmoser (E-Mail: david.breitenmoser@psi.ch, ORCID: 0000-0003-0339-6592)

The PDF includes:

Supplementary Figures 1–7
Supplementary Tables 1–4
Supplementary References

Contents

List of Supplementary Figures SIII

List of Supplementary Tables SIV

S1 Supplementary Figures S1

S2 Supplementary Tables S8

Supplementary References S12

List of Supplementary Figures

1 Background measurements S1
2 Trace and convergence plots for the dataset HF_Cs_1s S2
3 Trace and convergence plots for the dataset HF_Cs_5s S3
4 Trace and convergence plots for the dataset HF_Cs_5m S4
5 Trace and convergence plots for the dataset HF_Ba_1s S5
6 Trace and convergence plots for the dataset HF_Ba_5s S6
7 Trace and convergence plots for the dataset HF_Ba_5m S7

List of Supplementary Tables

1 Radiation measurement meta-data . . . S8
2 Prior distribution summary . . . S9
3 Posterior distribution summary for the datasets HF_Cs_1s, HF_Cs_5s, and HF_Cs_5m . . S10
4 Posterior distribution summary for the datasets HF_Ba_1s, HF_Ba_5s, and HF_Ba_5m . . S11

S1 Supplementary Figures

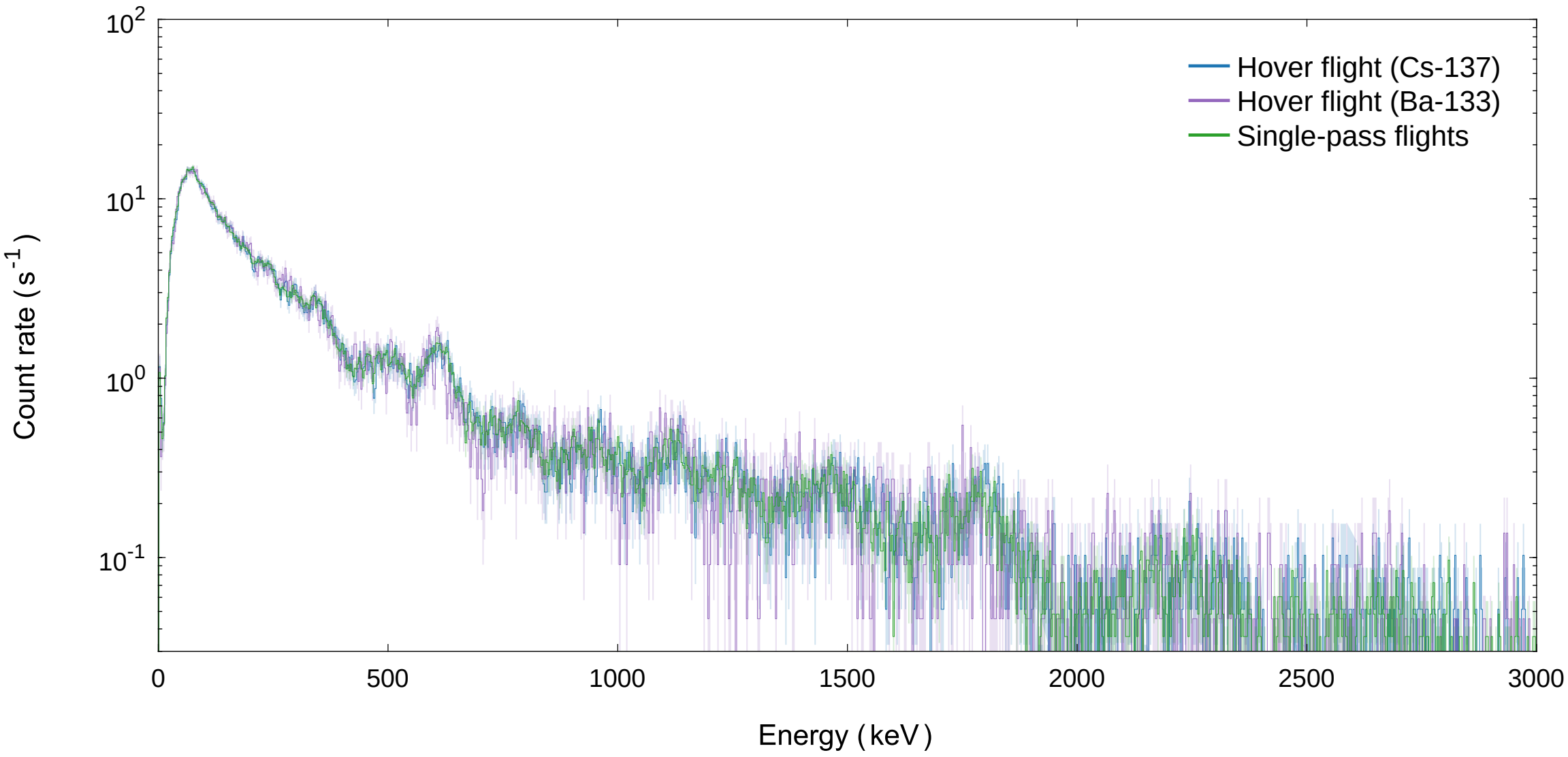

Supplementary Figure 1 Background measurements. Here, we display the measured background pulse-height spectra as a function of energy with an energy bin width of ~3 keV. The spectra were acquired by the Swiss Airborne Gamma-Ray Spectrometry (SAGRS) system [1] in the morning and afternoon on June 16, 2022 over Lake Thun during the ARM22 validation campaign [2] at orthometric heights equivalent to the ones of the hover and single-pass flight measurements utilized in the main study. Uncertainties are provided as 1 standard error shaded areas.

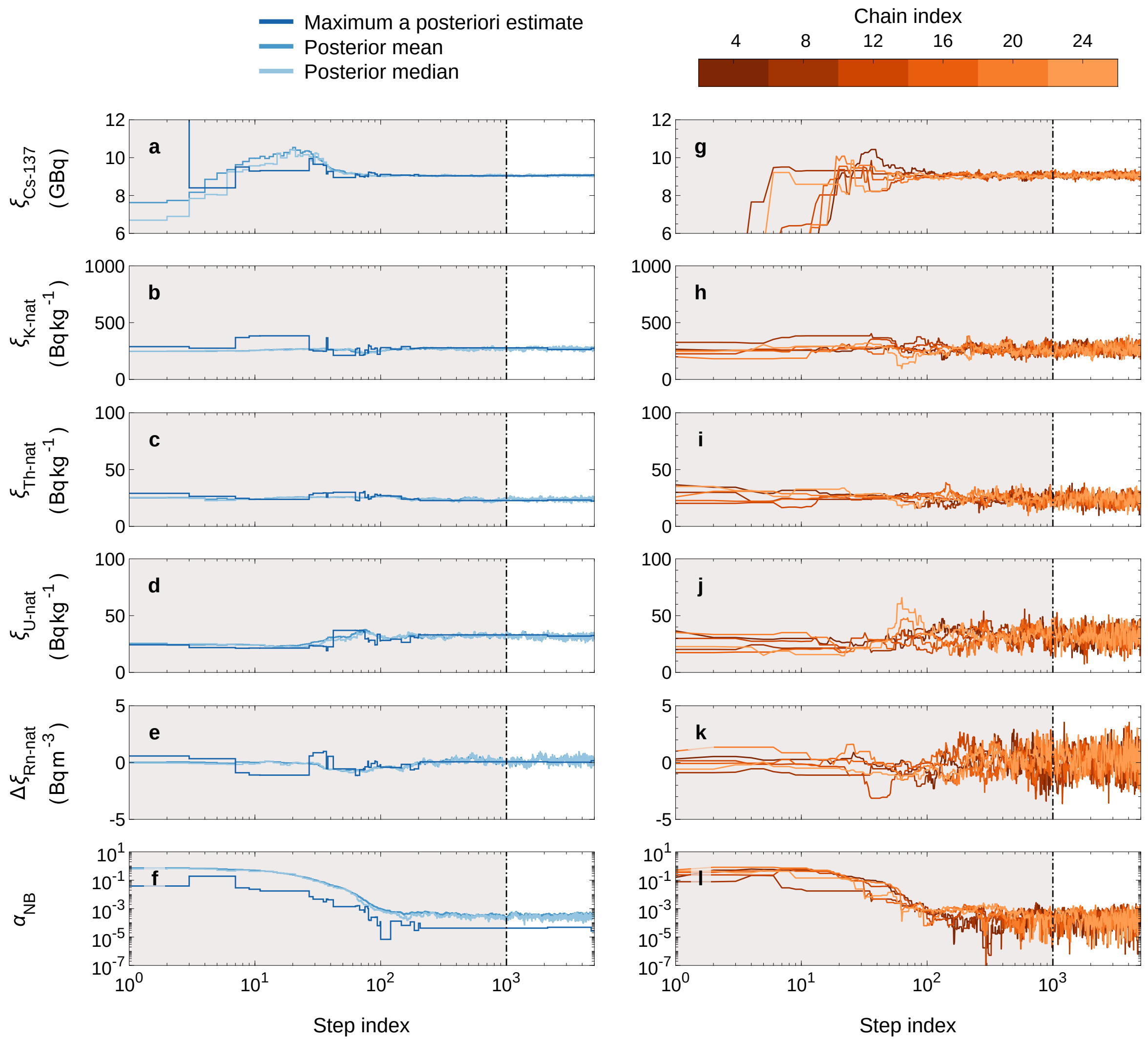

Supplementary Figure 2 Trace and convergence plots for the dataset `HF_Cs_1s`**.** These graphs present the convergence (**a**–**f**) and trace (**g**–**l**) plots for the Markov chain Monte Carlo (MCMC) based Bayesian inversion computations for the dataset `HF_Cs_1s`. The graphs are displayed as a function of the MCMC steps for each model parameter, i.e., the source strength of the sealed $^{137}_{55}$Cs point source ($\xi_{\text{Cs-137}}$), the source strengths of the three natural terrestrial radionuclides K_{nat}, Th_{nat} and U_{nat} ($\xi_{\text{K-nat}}$, $\xi_{\text{Th-nat}}$, $\xi_{\text{U-nat}}$), the source strength of the radon source term ($\Delta\xi_{\text{Rn-nat}}$) and the dispersion parameter of the negative binomial distribution (α_{NB}). The convergence is shown for the maximum a posteriori estimate, the posterior mean, and the posterior median. The trace plots display a subset of 6 out of the 24 simulated MCMC chains. The burn-in phase is highlighted for all subgraphs as gray-shaded areas with the related threshold marked by the dashed-dotted black line.

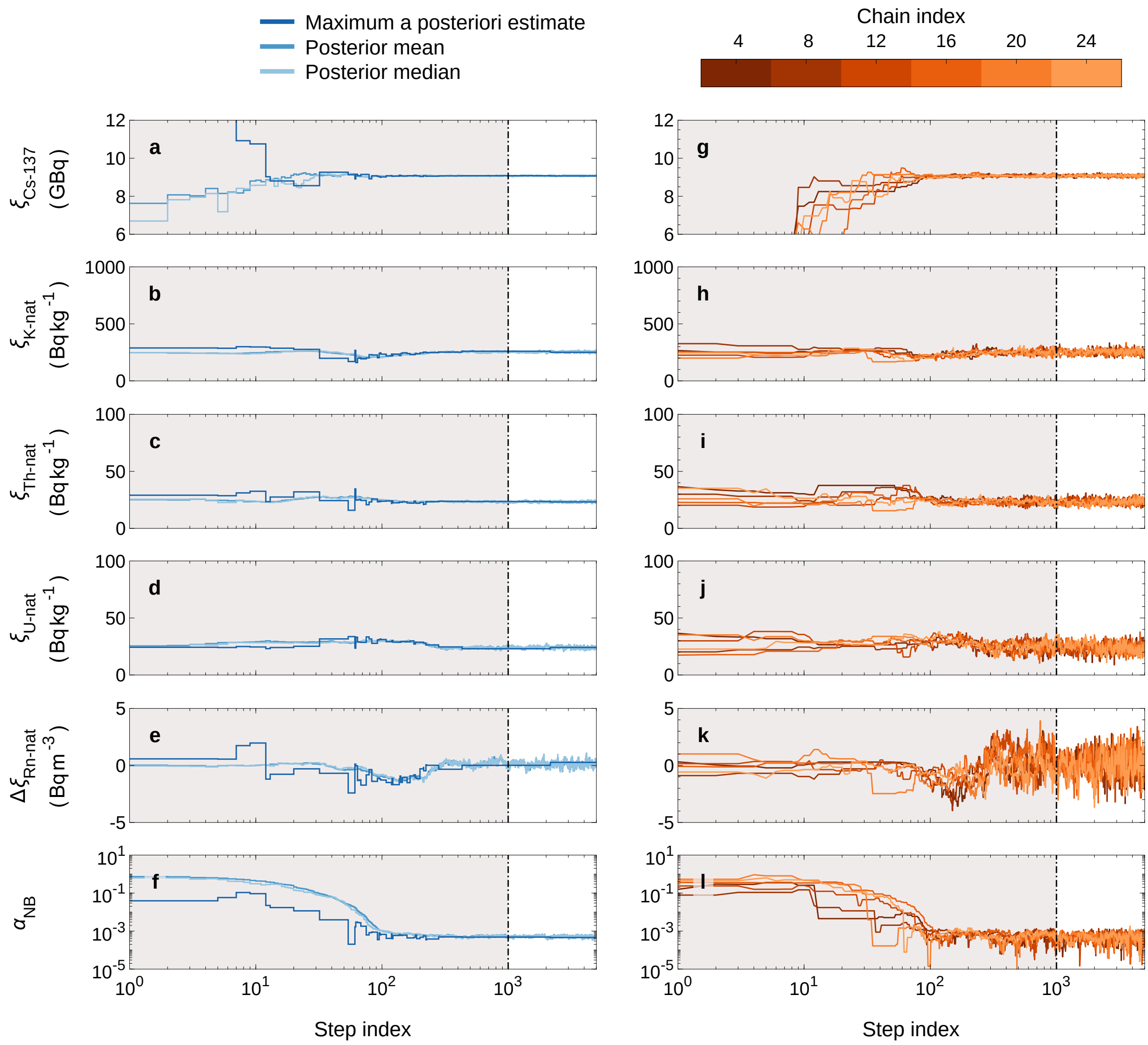

Supplementary Figure 3 Trace and convergence plots for the dataset HF_Cs_5s. Same as Supplementary Fig. 2, but using dataset HF_Cs_5s instead of dataset HF_Cs_1s.

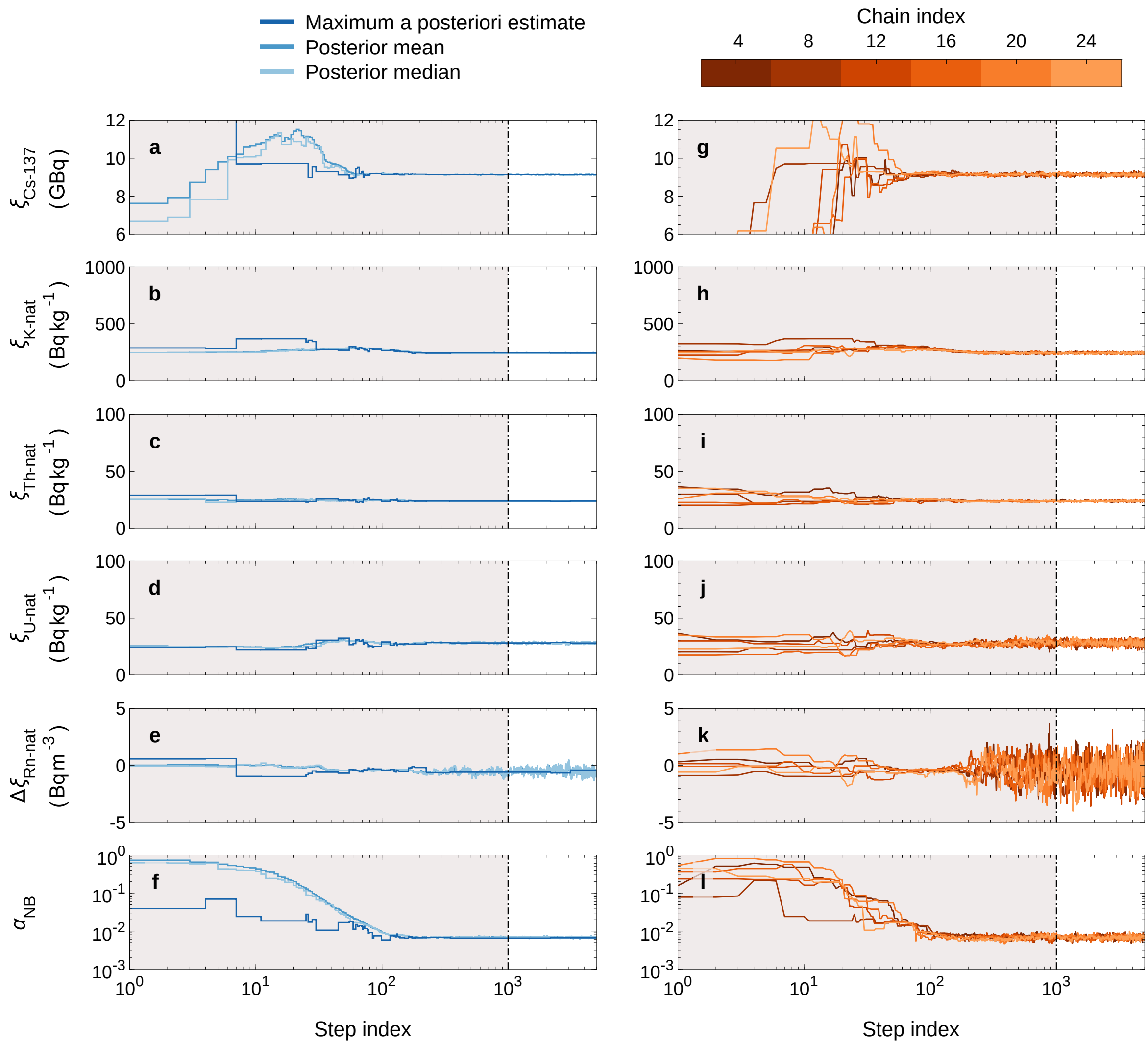

Supplementary Figure 4 Trace and convergence plots for the dataset HF_Cs_5m. Same as Supplementary Fig. 2, but using dataset HF_Cs_5m instead of dataset HF_Cs_1s.

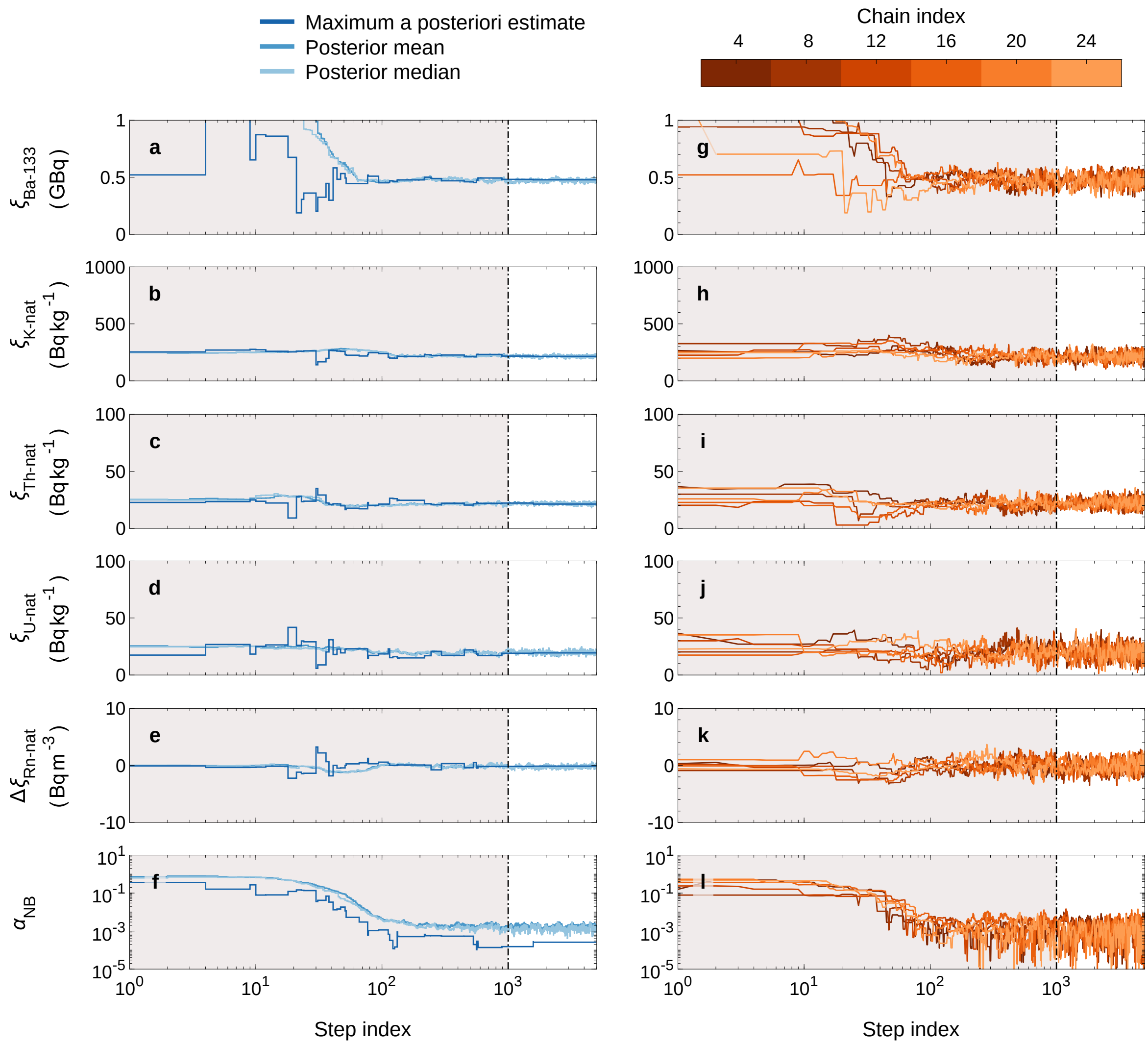

Supplementary Figure 5 Trace and convergence plots for the dataset HF_Ba_1s. Same as Supplementary Fig. 2, but using dataset HF_Ba_1s instead of dataset HF_Cs_1s.

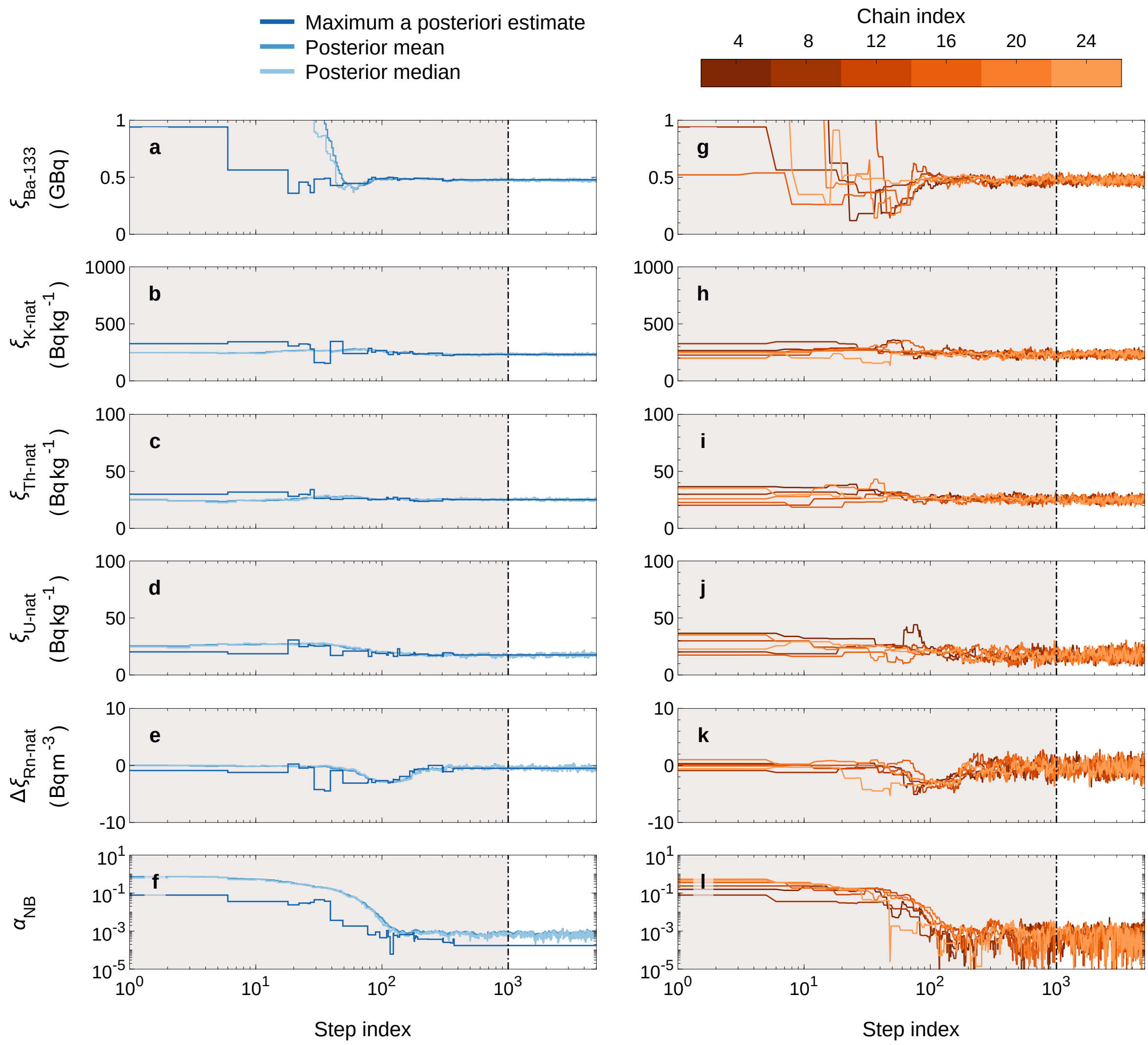

Supplementary Figure 6 Trace and convergence plots for the dataset HF_Ba_5s. Same as Supplementary Fig. 2, but using dataset HF_Ba_5s instead of dataset HF_Cs_1s.

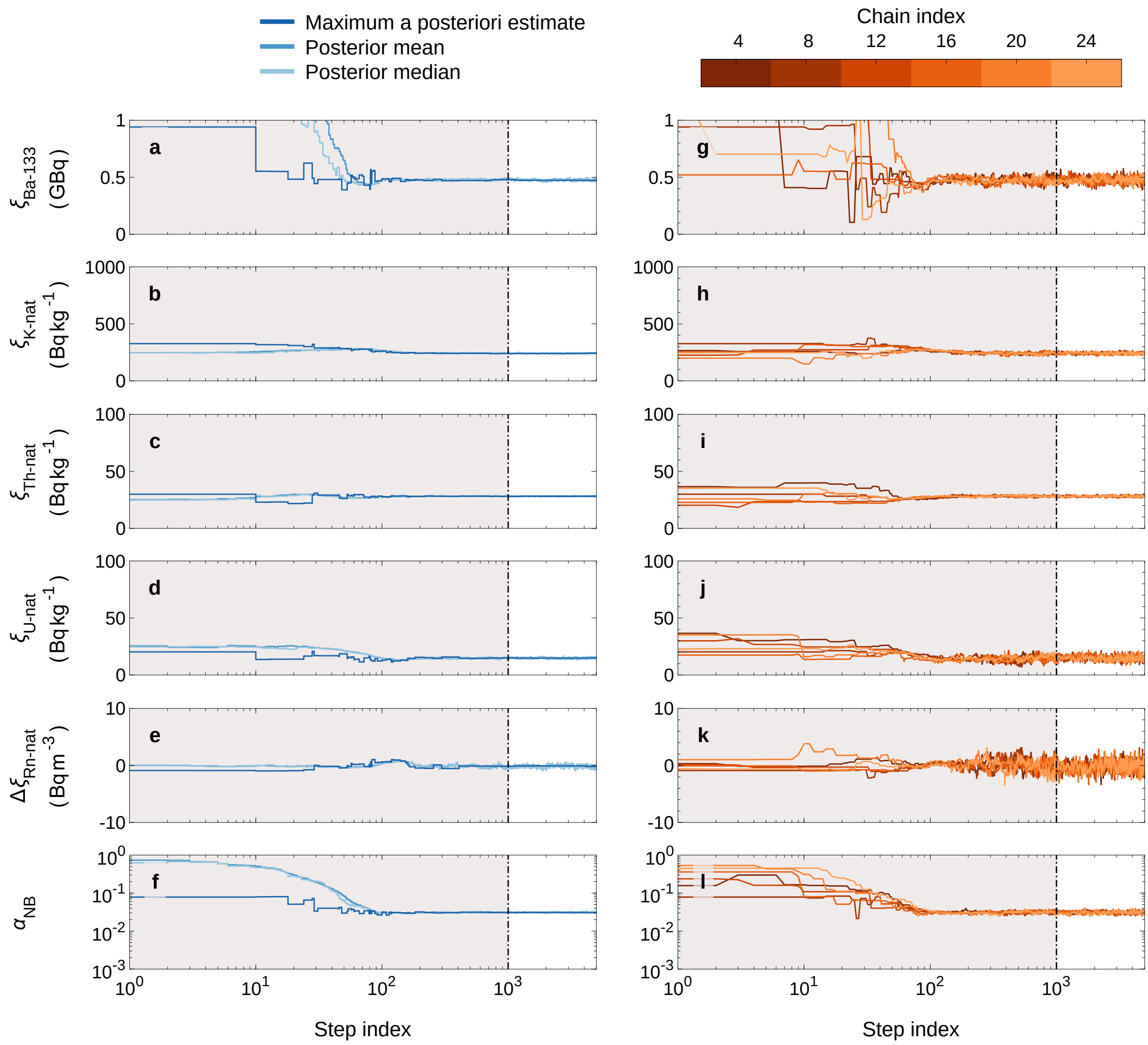

Supplementary Figure 7 Trace and convergence plots for the dataset HF_Ba_5m. Same as Supplementary Fig. 2, but using dataset HF_Ba_5m instead of dataset HF_Cs_1s.

S2 Supplementary Tables

Supplementary Table 1 **Radiation measurement meta-data.** Here, we present an overview of the radiation measurement datasets for the hover-flight (HF) and single-pass (SP) flight modes, highlighting deployed radionuclide sources alongside auxiliary sensor data. Provided uncertainties represent the standard error for the measurement (coverage factor $k = 1$) in least significant figure notation. Please refer to Ref. [1] for a detailed description of the associated radiation measurements and related data postprocessing. For the single-pass (SP) flights, we provide mean sensor values, while adopting time-resolved values for the Bayesian inference presented in the main study.

Identifier	Source⋆	$\mathscr{A}$• [GBq]	h_{air}∘ [m]	-α'† [°]	β'‡ [°]	γ'§ [°]	$\varrho_{fuel}$$ [%]	T_{air}# [°C]	p_{air}•• [hPa]	RH_{air}∘∘ [%]	t_{gr}†† [s]	t_b‡‡ [s]
HF_Ba_1s	$^{133}_{56}$Ba	0.47(2)	93(1)	316(3)	1(1)	−0.2(1)	94(2)	28.9(1)	953.9(1)	39.0(1)	1	22
HF_Ba_5s	$^{133}_{56}$Ba	0.47(2)	92(1)	314(3)	−0.33(5)	−1.5(1)	94(2)	28.9(1)	953.9(1)	39.0(1)	5	22
HF_Ba_5m	$^{133}_{56}$Ba	0.47(2)	90.8(1)	314(3)	−0.34(4)	−1.3(1)	94(2)	28.9(1)	953.9(1)	39.0(1)	296	22
HF_Cs_1s	$^{137}_{55}$Cs	9.0(5)	88(2)	142(3)	0.6(1)	−2.6(1)	57(2)	22.6(1)	954.6(1)	61.9(1)	1	39
HF_Cs_5s	$^{137}_{55}$Cs	9.0(5)	91(2)	142(3)	0.6(1)	−3.0(1)	57(2)	22.6(1)	954.6(1)	61.9(1)	5	39
HF_Cs_5m	$^{137}_{55}$Cs	9.0(5)	91.6(2)	142(3)	0.6(1)	−2.9(1)	57(2)	22.6(1)	954.6(1)	61.9(1)	232	39
SP_Ba	$^{133}_{56}$Ba	0.47(2)	30.6(1)	53(3)	1.9(2)	2.7(3)	31(2)	25.0(1)	954.7(1)	58.2(1)	6	83
SP_Cs	$^{137}_{55}$Cs	9.0(5)	30.5(1)	54(3)	1.2(3)	2.0(1)	45(2)	24.0(1)	954.6(1)	58.6(1)	6	83
SP_BaCs	$^{133}_{56}$Ba	0.47(2)	30.8(1)	58(3)	0.5(1)	3.2(1)	39(2)	25.0(1)	954.7(1)	55.4(1)	6	83
	$^{137}_{55}$Cs	9.0(5)										

⋆ Radionuclide source adopted in the corresponding gross measurement.
• Mean source activity.
∘ Mean ground clearance of the gross measurement determined by the radar altimeter of the AS332M1 helicopter. Note that the data was corrected for the vertical displacement between the radar altimeter, located at the bottom of the tail, and the spectrometer, mounted in the cargo bay (see Ref. [1]).
† Mean negative yaw angle relative to geographic north.
‡ Mean roll angle.
§ Mean pitch angle.
$ Mean fuel volume fraction. For the Monte Carlo simulations, the individual fuel volume fractions of the six tanks were adopted (see Ref. [1]). Here, we report only the average fraction across all tanks.
# Mean air temperature.
•• Mean air pressure.
∘∘ Mean relative humidity.
†† Gross measurement live time (rounded to seconds).
‡‡ Background measurement live time (rounded to seconds).

Supplementary Table 2 Prior distribution summary. This table summarizes the marginal prior distributions adopted for the Bayesian inference in the main study.

Variable⋆	Prior•	Prior parameters		Truncation	Unit
$\xi_{\text{Cs-137}}$	$\mathcal{N}$	$\mu = 10^9$	$\sigma = 10^{10}$	$[0, \infty]$	Bq
$\xi_{\text{Ba-133}}$	$\mathcal{N}$	$\mu = 10^9$	$\sigma = 10^{10}$	$[0, \infty]$	Bq
$\xi_{\text{K-nat}}$	$\mathcal{N}$	$\mu = 250$	$\sigma = 50$	$[0, \infty]$	Bq kg^{-1}
$\xi_{\text{Th-nat}}$	$\mathcal{N}$	$\mu = 25$	$\sigma = 10$	$[0, \infty]$	Bq kg^{-1}
$\xi_{\text{U-nat}}$	$\mathcal{N}$	$\mu = 25$	$\sigma = 10$	$[0, \infty]$	Bq kg^{-1}
$\Delta\xi_{\text{Rn-nat}}$	$\mathcal{N}$	$\mu = 0$	$\sigma = 1$	$[-\infty, \infty]$	Bq m^{-3}
α_{NB}	$\mathcal{N}$	$\mu = 0$	$\sigma = 1$	$[0, \infty]$	
x	$\mathcal{U}$	$l = \min x$	$u = \max x$	$[-\infty, \infty]$	m

⋆ Model parameters considered in the Bayesian inference, i.e., , the source strengths of the sealed $^{137}_{55}\text{Cs}$ and $^{133}_{56}\text{Ba}$ point sources ($\xi_{\text{Cs-137}}$, $\xi_{\text{Ba-133}}$), the source strengths of the three natural terrestrial radionuclides K_{nat}, Th_{nat} and U_{nat} ($\xi_{\text{K-nat}}$, $\xi_{\text{Th-nat}}$, $\xi_{\text{U-nat}}$), the source strength of the radon source term ($\Delta\xi_{\text{Rn-nat}}$), the dispersion parameter of the negative binomial distribution (α_{NB}), and the along-track position (x) in the single-pass measurements.

• With $\mathcal{N}$, we refer to the truncated univariate normal distribution $\mathcal{N}(\mu, \sigma, \theta_l, \theta_u)$ with mean μ, standard deviation σ and truncation $\theta \in [\theta_l, \theta_u]$, parametrized by the lower and upper boundary parameters θ_l and θ_u. With $\mathcal{U}$, we refer to the uniform distribution $\mathcal{U}(l, u)$ with lower threshold l and upper threshold u.

Supplementary Table 3 Posterior distribution summary for the datasets HF_Cs_1s, HF_Cs_5s, and HF_Cs_5m. This table includes posterior point and dispersion estimators for the datasets HF_Cs_1s, HF_Cs_5s, and HF_Cs_5m discussed in the main study. The listed estimators are the maximum a posteriori estimate (θ_{MAP}), the posterior mean (θ_{Mean}), and the posterior median (θ_{Median}) together with the 95 % credible interval and the posterior standard deviation (σ_θ). All numerical values displayed are rounded to three significant digits.

ID⋆	Variable•	θ_{MAP}	θ_{Mean}	θ_{Median}	95 % credible interval∘	σ_θ	Unit
HF_Cs_1s	$\xi_{Cs\text{-}137}$	9.08×10^{9}	9.06×10^{9}	9.06×10^{9}	$[8.88, 9.25] \times 10^{9}$	9.27×10^{7}	Bq
	$\xi_{K\text{-}nat}$	2.75×10^{2}	2.71×10^{2}	2.71×10^{2}	$[2.06, 3.38] \times 10^{2}$	3.39×10^{1}	$Bq\,kg^{-1}$
	$\xi_{Th\text{-}nat}$	2.23×10^{1}	2.38×10^{1}	2.38×10^{1}	$[1.60, 3.20] \times 10^{1}$	4.13	$Bq\,kg^{-1}$
	$\xi_{U\text{-}nat}$	3.25×10^{1}	3.16×10^{1}	3.16×10^{1}	$[1.90, 4.38] \times 10^{1}$	6.35	$Bq\,kg^{-1}$
	$\Delta\xi_{Rn\text{-}nat}$	1.61×10^{-1}	1.74×10^{-1}	1.81×10^{-1}	$[-1.77, 2.04]$	9.75×10^{-1}	$Bq\,m^{-3}$
	α_{NB}	2.66×10^{-5}	3.59×10^{-4}	2.60×10^{-4}	$[1.06, 128] \times 10^{-5}$	3.38×10^{-4}	
HF_Cs_5s	$\xi_{Cs\text{-}137}$	9.07×10^{9}	9.08×10^{9}	9.08×10^{9}	$[8.98, 9.17] \times 10^{9}$	4.80×10^{7}	Bq
	$\xi_{K\text{-}nat}$	2.51×10^{2}	2.54×10^{2}	2.54×10^{2}	$[2.14, 2.95] \times 10^{2}$	2.08×10^{1}	$Bq\,kg^{-1}$
	$\xi_{Th\text{-}nat}$	2.32×10^{1}	2.33×10^{1}	2.32×10^{1}	$[1.94, 2.74] \times 10^{1}$	2.05	$Bq\,kg^{-1}$
	$\xi_{U\text{-}nat}$	2.42×10^{1}	2.40×10^{1}	2.41×10^{1}	$[1.65, 3.17] \times 10^{1}$	3.91	$Bq\,kg^{-1}$
	$\Delta\xi_{Rn\text{-}nat}$	2.73×10^{-1}	1.26×10^{-1}	1.27×10^{-1}	$[-1.80, 2.05]$	9.77×10^{-1}	$Bq\,m^{-3}$
	α_{NB}	4.58×10^{-4}	5.32×10^{-4}	5.12×10^{-4}	$[1.42, 10.3] \times 10^{-4}$	2.27×10^{-4}	
HF_Cs_5m	$\xi_{Cs\text{-}137}$	9.15×10^{9}	9.14×10^{9}	9.14×10^{9}	$[9.03, 9.26] \times 10^{9}$	5.86×10^{7}	Bq
	$\xi_{K\text{-}nat}$	2.45×10^{2}	2.45×10^{2}	2.45×10^{2}	$[2.34, 2.56] \times 10^{2}$	5.58	$Bq\,kg^{-1}$
	$\xi_{Th\text{-}nat}$	2.39×10^{1}	2.39×10^{1}	2.39×10^{1}	$[2.31, 2.48] \times 10^{1}$	4.18×10^{-1}	$Bq\,kg^{-1}$
	$\xi_{U\text{-}nat}$	2.81×10^{1}	2.81×10^{1}	2.81×10^{1}	$[2.44, 3.19] \times 10^{1}$	1.95	$Bq\,kg^{-1}$
	$\Delta\xi_{Rn\text{-}nat}$	-4.11×10^{-1}	-5.04×10^{-1}	-4.92×10^{-1}	$[-2.40, 1.36]$	9.62×10^{-1}	$Bq\,m^{-3}$
	α_{NB}	6.65×10^{-3}	6.87×10^{-3}	6.83×10^{-3}	$[5.54, 8.43] \times 10^{-3}$	7.33×10^{-4}	

⋆ Dataset identifier.

• Model parameters θ considered in the Bayesian inference, i.e., the source strength of the sealed $^{137}_{55}Cs$ point source ($\xi_{Cs\text{-}137}$), the source strengths of the three natural terrestrial radionuclides K_{nat}, Th_{nat}, and U_{nat} ($\xi_{K\text{-}nat}$, $\xi_{Th\text{-}nat}$, $\xi_{U\text{-}nat}$), the source strength of the radon source term ($\Delta\xi_{Rn\text{-}nat}$), and the dispersion parameter of the negative binomial distribution (α_{NB}).

∘ Central credible interval with a probability mass of 95 %.

Supplementary Table 4 Posterior distribution summary for the datasets HF_Ba_1s, HF_-Ba_5s, and HF_Ba_5m. The Same as Supplementary Table 3, but using the datasets HF_Ba_1s, HF_Ba_5s, and HF_Ba_5m instead of the datasets HF_Cs_1s, HF_Cs_5s, and HF_Cs_5m.

ID⋆	Variable•	θ_{MAP}	θ_{Mean}	θ_{Median}	95 % credible interval∘	σ_θ	Unit
HF_Ba_1s	$\xi_{Ba\text{-}133}$	4.78×10^{8}	4.71×10^{8}	4.72×10^{8}	$[3.80, 5.59] \times 10^{8}$	4.54×10^{7}	Bq
	$\xi_{K\text{-}nat}$	2.15×10^{2}	2.16×10^{2}	2.16×10^{2}	$[1.52, 2.83] \times 10^{2}$	3.36×10^{1}	$Bq\,kg^{-1}$
	$\xi_{Th\text{-}nat}$	2.13×10^{1}	2.18×10^{1}	2.18×10^{1}	$[1.49, 2.91] \times 10^{1}$	3.61	$Bq\,kg^{-1}$
	$\xi_{U\text{-}nat}$	1.94×10^{1}	1.98×10^{1}	1.99×10^{1}	[8.58, 30.7]	5.62	$Bq\,kg^{-1}$
	$\Delta\xi_{Rn\text{-}nat}$	-1.20×10^{-1}	-2.10×10^{-1}	-2.21×10^{-1}	[−2.10, 1.75]	9.82×10^{-1}	$Bq\,m^{-3}$
	α_{NB}	2.59×10^{-4}	1.88×10^{-3}	1.33×10^{-3}	$[5.44, 656] \times 10^{-5}$	1.78×10^{-3}	
HF_Ba_5s	$\xi_{Ba\text{-}133}$	4.81×10^{8}	4.74×10^{8}	4.74×10^{8}	$[4.31, 5.18] \times 10^{8}$	2.24×10^{7}	Bq
	$\xi_{K\text{-}nat}$	2.36×10^{2}	2.32×10^{2}	2.32×10^{2}	$[1.96, 2.70] \times 10^{2}$	1.87×10^{1}	$Bq\,kg^{-1}$
	$\xi_{Th\text{-}nat}$	2.50×10^{1}	2.52×10^{1}	2.52×10^{1}	$[2.13, 2.93] \times 10^{1}$	2.06	$Bq\,kg^{-1}$
	$\xi_{U\text{-}nat}$	1.68×10^{1}	1.76×10^{1}	1.76×10^{1}	$[1.05, 2.49] \times 10^{1}$	3.69	$Bq\,kg^{-1}$
	$\Delta\xi_{Rn\text{-}nat}$	-1.72×10^{-1}	-4.45×10^{-1}	-4.55×10^{-1}	[−2.32, 1.46]	9.63×10^{-1}	$Bq\,m^{-3}$
	α_{NB}	5.59×10^{-5}	7.77×10^{-4}	6.26×10^{-4}	$[3.16, 237] \times 10^{-5}$	6.34×10^{-4}	
HF_Ba_5m	$\xi_{Ba\text{-}133}$	4.73×10^{8}	4.76×10^{8}	4.76×10^{8}	$[4.29, 5.26] \times 10^{8}$	2.47×10^{7}	Bq
	$\xi_{K\text{-}nat}$	2.43×10^{2}	2.42×10^{2}	2.42×10^{2}	$[2.26, 2.58] \times 10^{2}$	8.29	$Bq\,kg^{-1}$
	$\xi_{Th\text{-}nat}$	2.82×10^{1}	2.82×10^{1}	2.82×10^{1}	$[2.71, 2.93] \times 10^{1}$	5.81×10^{-1}	$Bq\,kg^{-1}$
	$\xi_{U\text{-}nat}$	1.49×10^{1}	1.49×10^{1}	1.49×10^{1}	$[1.09, 1.91] \times 10^{1}$	2.10	$Bq\,kg^{-1}$
	$\Delta\xi_{Rn\text{-}nat}$	-1.73×10^{-1}	-1.67×10^{-1}	-1.56×10^{-1}	[−2.04, 1.67]	9.49×10^{-1}	$Bq\,m^{-3}$
	α_{NB}	3.06×10^{-2}	3.11×10^{-2}	3.10×10^{-2}	$[2.69, 3.58] \times 10^{-2}$	2.27×10^{-3}	

⋆ Dataset identifier.

• Model parameters θ considered in the Bayesian inference, i.e., the source strength of the sealed $^{133}_{56}Ba$ point source ($\xi_{Ba\text{-}133}$), the source strengths of the three natural terrestrial radionuclides K_{nat}, Th_{nat}, and U_{nat} ($\xi_{K\text{-}nat}$, $\xi_{Th\text{-}nat}$, $\xi_{U\text{-}nat}$), the source strength of the radon source term ($\Delta\xi_{Rn\text{-}nat}$), and the dispersion parameter of the negative binomial distribution (α_{NB}).

∘ Central credible interval with a probability mass of 95 %.

Supplementary References

1. Breitenmoser, D., Stabilini, A., Kasprzak, M. M. & Mayer, S. Development and Validation of a High-Fidelity Full-Spectrum Monte Carlo Model for the Swiss Airborne Gamma-Ray Spectrometry System. *Nuclear Instruments and Methods in Physics Research Section A: Accelerators, Spectrometers, Detectors and Associated Equipment* **1077,** 170512. doi:10.1016/j.nima.2025.170512. arXiv: 2502.02102 (Aug. 1, 2025).
2. Butterweck, G. *et al. Aeroradiometric Measurements in the Framework of the Swiss Exercise ARM22* (Paul Scherrer Institut (PSI), Villigen PSI, Switzerland, 2023). doi:10.55402/psi:51194.
