# Supplementary material for: Quantitative mobile gamma-ray spectrometry through Bayesian inference
Source: Nat Commun. 2026 Oct 26;17:10913. doi: 10.1038/s41467-026-78464-2 (PMC13710540; doi:10.1038/s41467-026-78464-2)
Supplement: Supplementary file 2 [file 41467_2026_78464_MOESM2_ESM.pdf]

nature portfolio

# Peer Review File

# Quantitative mobile gamma-ray spectrometry through Bayesian inference

Corresponding Author: Dr David Breitenmoser

**This file contains all reviewer reports in order by version, followed by all author rebuttals in order by version.**

Version 0:

Reviewer comments:

Reviewer #1

(Remarks to the Author)
Key Results:

The authors describe a Bayesian inference framework designed to estimate radionuclide activity using data from mobile gamma-ray spectrometry (MGRS) systems. The framework was validated using a helicopter-borne system in a static hover at a 90-meter standoff over two separate radioactive sources (137Cs and 133Ba). By analyzing single integrated spectra over three distinct dwell times (1s, 5s, and 5min). The authors calculated posterior distributions based on a six-parameter model: one anthropogenic source activity, three natural terrestrial backgrounds (K, U, Th), one atmospheric Radon component, and a Negative Binomial overdispersion parameter (α). To support this, extensive simulations were conducted to model both the expected gamma-ray flux and the detector response, accounting for attenuation and scattering through detailed helicopter mass models. The results indicate that the true source activity consistently falls within the 68% credible interval of the posterior distribution for both sources.

Validity:

The authors have been impressively thorough in their modeling and evaluation. The data appear consistent with the proposed model, and the Bayesian framework is mathematically sound and well-founded for this application.

Significance:

The presented Bayesian framework is novel and highly relevant for mobile gamma-ray spectrometry, with the potential for broad application. The modeling effort and the rigor of the data analysis are commendable. However, the scope of the presented data and analysis is somewhat limited. While the modeling is sophisticated, the experimental validation is relatively simple, consisting of single spectra measured in a highly controlled environment with minimal variability.

As such the title and abstract is slightly inaccurate. The authors do not present any "mobile" results, the data where collected in a purely static configuration. A primary strength of an MGRS system is its ability to exploit sensitivity modulations caused by attenuation effects as the detector moves through space; this advantage is lost when data are aggregated as aggressively as done here. Furthermore, the authors assume known source-to-detector orientation and separation. Under such constrained conditions, most frameworks should yield correct results. The practical challenge in MGRS is typically the detection and localization of unknown sources. Incorporating source location as an additional three parameters would significantly enhance the significance but would require dynamic datasets where the platform is not static. Additionally, the model assumes a known emission profile and environmental transport. Unconstrained real-world environments, such as sources in unknown shielding or of unknown types, will not be tractable with simulations alone. While this study is an excellent technical endeavor, the data analysis is limited in scope. The editors might evaluate whether this narrow focus is more suited for Scientific Reports than the broader platform of Nature Communications.

Data and Methodology:

The quality of the approach, and presentation is exceptional. It would be interesting seeing the method being applied to more complex datasets involving moving systems, sensitivity modulation through detector separation, and limited a priori

knowledge of the environment. The authors released the spectral data and simulation code. As is, the results won't be easily reproducible. For full reproducibility they should release the detector response, source and background emission models, and source templates that were used. Optimally, they would also release the code that performs the Bayesian inference.

Analytical Approach:

The analytical approach is impressive and the Bayesian framework is technically interesting. The primary strength lies in the inclusion of the dispersion parameter (α), which is a truly novel aspect that is difficult to replicate in a Frequentist framework. While perhaps not entirely novel in the broader context of Bayesian theory, its application here is a significant highlight.

Suggested Improvements:

As previously noted, this work is exceptional. The following suggestions are intended to challenge the authors to further refine their publication such that they can meet the rigorous standards of top tier journal.

1. Introduction

The introduction focuses heavily on planetary and space-based applications and dynamic systems, whereas the analysis is restricted to a static dataset. The authors should clarify this relationship or provide a more focused introduction relevant to helicopter-based surveys.

Line 15: "dynamically varying source–detector geometries" is slightly inaccurate. Typically, the geometries of the source and detector themselves do not vary (unless dealing with dispersed sources); rather, their positions vary relative to each other and the environment.

Line 27: "empirical calibration libraries" is not clearly defined.

Line 34: The claim that approximations fail to reproduce platform-dependent scattering/attenuation is contradicted by Ref. 9, which performs similar modeling.

Line 45 "overdispersion effects that arise from fluctuating experimental conditions": To ensure clarity, the authors should expand on the physical mechanism driving this. It appears the overdispersion stems from environmental fluctuations occurring on timescales different from those the model accounts for, specifically, where a time-varying integrated probability of detection results in non-Poissonian statistical properties.

Line 46: The claim that Poisson-based MLE provides "no rigorous framework for uncertainty quantification" is inaccurate. Rigorous UQ is established in several works (e.g., Ref. 7, 42, https://doi.org/10.1109/TNS.2020.3024735, https://doi.org/10.1109/TNS.2022.3226707, https://doi.org/10.48550/arXiv.2412.02926, https://doi.org/10.1038/s41598-024-71336-z.

2. Results

Section 2.1: This section would benefit from simplified paraphrasing for less experienced readers. For example, defining vector(y)i as gamma-ray histograms, curly d_k as environmental parameters, and theta as the unknowns (activities). The distinction between theta and xi should also be clarified.

Line 68: "pulse-height spectral data". It remains unclear if the authors worked in pulse-height (channel) space or energy space; Figure 2 suggests the latter.

Line 72: "single point solution" is potentially confusing; "point estimate and confidence interval" are the standard Frequentist terms and both are available in a Frequentist framework.

Line 75 equation: The y-subscript for "N in Natural Numbers" is missing. While, consistent, the use of N_curly(y) for both the curly Y definition but also the curly D definition is a bit confusing.Define it first as the number of measurement configurations.

Line 87: "nuisance gamma-ray background". The term "nuisance" is unusual here when referring to cosmic and secondary effects. It is not a nuisance parameter in the Bayesian sense either.

Line 106: "multiscale". The authors should clarify what is meant by "multiscale" in this context.

Section 2.2: The gain of NaI(Tl) detectors is temperature-sensitive. The authors should note how gain stabilization was maintained (e.g., environmental control in the helicopter bay).

Line 130-136: "RLLSpec". I am unclear if RLLSpec was used for data normalization, as this would violate the count-space requirements of the framework. Also the missing discussion of energy calibration makes me believe the authors mean RLLCal?

Section 2.3:

Line 157: "double-differential flux banks" is an abstract term and requires a brief definition.

Line 163: Radon is treated as both a nuisance source and a fit parameter; this distinction should be explicitly noted.

Line 172: It would be prudent to mention here that the probabilistic model used is a Negative Binomial distribution.

Section 2.4: While the exact parameters for the prior are provided in the supplementary materials, the choice of prior should be mentioned here and the reason for this choice outlined. The Prior is shown in Fig. 2, but there is no mention of it anywhere in the main text.

Figure 2: The "Prior" (grey) is difficult to see and should be darkened. The K-40 line appears wider on the right; the authors should clarify if this is a plotting artifact. I would strongly suggest including residual plots (deviance or squared) to support the claim of model alignment.

Line 202 - 206: The interpretation of elevated overdispersion at longer sampling times feels like conjecture. It is more likely that the model is simply not accurate enough to account for second-order effects (e.g., sky-shine, back-scattering, or mass model inaccuracies) at 5 min integration time. If the authors believe their model is sufficiently accurate to describe the data at that level of fidelity they should provide proof or references.

Section 2.5:

Line 221: Accuracy is reported by comparing posterior medians to reference activities. The authors should clarify if these "reference values" were measured or derived from data sheets.

Line 229-230: While radon seems stable, alpha certainly varies in width and location.

Line 230 - 232: This phrase is unclear. The authors talk about intrinsic limits, I believe the limits are the models accuracies, not the method.

Line 239 - 240: "statistically insignificant" deviations. As these sit near the 68% credible interval, they are not as credible as this sounds, which raises the possibility of systematic bias and missing second order effects.

Line 247: The authors mention reference values for terrestrial components. What are these reference values? If there are ground truth data, they should be also added to Fig 2.

3. Discussion

The authors should discuss the functional abilities and limits of the model in more detail: Can it detect the absence or presence of a source, identify isotopic composition of sources, detect multipe sources at once, or quantify distributed sources?

Line 252 - 253: The authors claim to prove the framework for "MGRS," but the study is technically limited to static GRS.

Line 255: This statement is too vague. Many publications correctly handle sparse counting statistics (albit in a Frequentist framework). It is also unclear to me what improvements the authors did to with respect to spectral template modeling.

Line 260 - 261: "realistic MGRS conditions". A static hover does not represent the background fluctuations and spectral dynamics of a single-pass survey.

Line 268 - 269: The blanket statement is not characterizing previous work accurately.

Line 276 - 277: Another blanket statement that is not substantiated. The Possion assumption doesn't bias the source-strength estimate. If biases are observed they are analysis or modeling artifacts.

LIne 280 - 281: Substantially stronger deviation from Poisson statistics are only expected if the model is insufficiently accurate to capture the data dynamics. It is not a statistical property.

4. Methods

Lines 359 - 361: This implies the simulation of pulse-height spectra in channel space. This should be clearly stated in the Results section, or clarified otherwise here.

Line 368: "2×109 primaries distributed across 102 independent runs". Please clarify what is meant by "primaries" and "runs."

Line 427 - 429: The fact that a Gaussian prior distribution was used should be mentioned here.

Clarity and context:

The text is well-written and very carefully and accurately phrased. It is very mathematical, in my opinion, to the extent that the

authors' true intentions are often hidden behind abstract and overly complex concepts. While the technical rigor is commendable, the high level of formal complexity may present a barrier to accessibility, even for experts in the field. This isn't a criticism of the depth itself, but a greater emphasis on the underlying intuition alongside the formalism would ensure the work's significant contributions are more easily absorbed. Overall, the use of the English language is exceptional.

References:

The list of references is extensive and complete. Inclusion of the planetary references only make sense if they are relevant for the Bayesian methodology.

Expertise:

While I am not a specialist in Bayesian modeling, the authors' presentation of the framework makes sense and seems accurate based on my assessment of their logic.

Reviewer #2

(Remarks to the Author)
This is a well-organized and well-presented work of scientific value in the field of aerial gamma-ray spectrometry survey. It represents an advance over traditional methods of spectral analysis and is reasonably well-situated within the context of other work in this area.

There are no issues with the science.

There is one issue with the way it is presented which is easily dealt with. The authors have concentrated on characterizing the energy spectrum and emission strengths of the radioisotope sources. These sources have other characteristics though; they have a location, they may have a shape and an extent, and this work does not present a quantification of these other attributes. Throughout, but especially in the abstract and introduction, the authors should be careful to specify when they talk about ‘quantifying the source’ that it is the radioisotope source strength, or the energy spectrum, that they are quantifying and it is not a full source-characterization quantification.

I have a number of specific comments for consideration by the authors. For example, the presentation of the figures can be improved, there are a few places where the explanation can be clearer, there are alternative ways to estimate uncertainty. I have a suggestion of a group to reach over to doing a related analysis, and have noted some variables which are not fully defined before first use. These minor comments I have annotated in the attached .pdf version of the manuscript.

Version 1:

Reviewer comments:

Reviewer #1

(Remarks to the Author)
The authors addressed most of my concerns in their rebuttal, and the addition of Section 2.4 considerably improves the quality of the presented work. As such, I believe it is now well aligned with the standards of Nature Communications.

I have a few major but subtle concerns left that I believe can be relatively easily addressed:

- The first concerns how the authors frame the motivation for their method. Throughout the paper (particularly in the Introduction and Discussion), the authors repeatedly argue for their Bayesian approach by claiming what classical Poisson or Gaussian MLE methods cannot do (stating that they produce biased estimates, yield unstable solutions, or fail with sparse statistics). However, because the authors do not actually run a direct side-by-side comparative benchmark against a standard frequentist/MLE pipeline on this dataset, these claims about the limitations of Poisson/MLE feel unsubstantiated. I agree that the presented Bayesian method is very powerful and well founded on its own terms. I suggest the authors reframe these statements: instead of claiming classical Poisson/MLE fails at X, simply point out what the Bayesian framework naturally provides (such as principled uncertainty quantification, direct access to parameter correlations, flexible handling of overdispersed likelihoods, and robust treatment of nuisance parameters).
The second is regarding $\alpha\_NB$ and how the authors argue its behavior. I believe what they observe has a simple explanation. Relative statistical uncertainties scale as $1/\sqrt{N}$, so at low statistics any modeling artifact (overdispersion, pileup, template inaccuracy) is simply buried in the noise. Once the statistics increase, the relative uncertainty drops quickly and tension with the model becomes measurable. I have seen this in my own work many times: at low statistics a model describes the data very well, but when the integration time is increased the fit degrades. $\alpha\_NB$ can absorb some of this tension, and at the 5 min integration time it is required to do so. I do not believe that small variations over 5 minutes lead to additional overdispersion, as implied at line 215, but rather that at 5 min there are sufficient statistics to sense any model deficiency. While the authors mostly follow this line of thought, I believe they can improve the language around it. In particular, the statement at lines 217–220 seems to argue the opposite. Modeling gamma-ray transport in unconstrained environments is excruciatingly hard, and this work does it about as well as I have seen. Clearly stating the limits of one's approach does not weaken a publication but strengthens it.

The last is about modeling uncertainties. The authors largely assume the model inputs are exact, while acknowledging elsewhere that the GNSS positioning, for example, carries non-negligible uncertainty. I recognize that handling these properly is well beyond the scope of the current work: they are not amenable to simple downstream error propagation, since they enter at the template-generation stage. It would be good, however, to state this limitation explicitly and adding full propagation of D's uncertainties to the Discussion as a potential direction for future work would also be reasonable.

Additionally, I have a few smaller concerns:

- Section 2.1 mentions that a template evaluation takes O(1) s, and line 114 gives $O(10^4)$ likelihood evaluations, but the resulting end-to-end runtime is never stated. It is important to provide context on whether an inference runs in minutes, hours, or days; please add a typical wall-clock runtime. The method is presented as much faster than traditional Monte Carlo approaches, but relies on $5.5 \times 10^4$ core-hours for the IRF set and $\sim 10^2$ core-hours per source for the flux libraries. The upfront cost is therefore high, comparable to what is quoted for a single brute-force evaluation, but the results are reusable, and that is worth mentioning as well.
- Section 4.3 needs to better discuss the flux scoring methodology. Furthermore, Ref. [40] appears not to include a ground plane, so it is necessary to be precise here about how the distance of the detector from the ground plane (30 m and 90 m) is accounted for, and whether it is generally assumed that the helicopter does not change altitude during the encounter.
- Section 4.4 describes four independently read-out crystals, but Section 2.2 mentions a single volume. Please simply state in Section 2.2 that the four independent crystals are effectively used as a single, spatially unresolved detector.
- The abstract could be improved slightly. First, I would carefully weigh the use of "sources" when referring to radioactive material in general. Second, I personally do not understand what "platform-dynamic" means. Third, you do not present source localization of distributed sources; one could argue that the backgrounds are distributed, but the phrasing implies something different. Fourth, the claim of ~1% error in 1 s is a bit strong given the results, which are more on the order of 2–4%. Finally, the statement that this method quantifies anthropogenic sources that conventional methods cannot resolve would require proof that they are indeed inaccessible to classical methods. The sources here are relatively strong and clearly visible in the spectra, so I do not think even the claim of particular sensitivity is substantiated. At minimum, please add a comma after "resolve", as the phrase is currently ambiguous regarding what the 1 s and ~1% error refer to.
- Section 2.1: It would help to explain the experimental conditions here rather than at lines 167–168, as the concept is otherwise a bit too abstract. The same is true for the source mixture S; you could explain here that this is simply an assumption about which and how many sources are present.
- Section 2.3 should state right in the first paragraph that the source location is not estimated here. It is currently only implied in the following paragraph.
- Overall, I would describe the dimensionality of the problem better. How many bins per spectrum are used, and how many spectra (1 or 6, I believe)? The data are quite limited, which makes the accuracy the more impressive.
- Panel c in Figs. 4–6 is extremely complex. A bit more context on how to interpret the various numbers would be valuable, and it may help to mention that the model index is always ordered from least likely to most likely.
- O(X) notation: I do not see why it is needed in most places, but if you wish to keep it, please use O(10) m, O(100) m, O(100) km, which conveys the scale directly. $O(10^5)$ m takes me 30 seconds to work out.
- Line 201: I think the best estimates and uncertainties should be added to Fig. 3 or mentioned here explicitly. Otherwise, the statement is not verifiable.
- There are two or three different notations for the natural radon component; please choose one.
- The posterior in Fig. 2 is a bit hard to see, and given that the templates are histograms, step functions could be used.

Finally, I would like to applaud the authors for carrying out such a challenging analysis with this level of rigor, and for producing a truly excellent publication. I am looking forward to seeing this work published.

Reviewer #2

(Remarks to the Author)
After reading the response of the authors to the reviewer's comments, I recommend proceeding with publication of this revised version of the manuscript.

**Open Access** This Peer Review File is licensed under a Creative Commons Attribution 4.0 International License, which permits use, sharing, adaptation, distribution and reproduction in any medium or format, as long as you give appropriate credit to the original author(s) and the source, provide a link to the Creative Commons license, and indicate if changes were made.
In cases where reviewers are anonymous, credit should be given to 'Anonymous Referee' and the source.
The images or other third party material in this Peer Review File are included in the article's Creative Commons license, unless indicated otherwise in a credit line to the material. If material is not included in the article's Creative Commons license and your intended use is not permitted by statutory regulation or exceeds the permitted use, you will need to obtain permission directly from the copyright holder.
To view a copy of this license, visit https://creativecommons.org/licenses/by/4.0/

# Peer Review Report for

## Quantitative mobile gamma-ray spectrometry through Bayesian inference

David Breitenmoser[1,2,*], Alberto Stabilini[1], Malgorzata Magdalena Kasprzak[1], Sabine Mayer[1]

[1]Department of Radiation Safety and Security, Paul Scherrer Institute (PSI), Forschungsstrasse 111, Villigen PSI, 5232, Switzerland

[2]Department of Nuclear Engineering & Radiological Sciences, University of Michigan, 2355 Bonisteel Blvd., Ann Arbor, MI 48109-2104, United States of America

[*]Lead and contact author: David Breitenmoser (E-Mail: david.breitenmoser@psi.ch, ORCID: 0000-0003-0339-6592)

## Preface

We would like to thank the referees and editor for their important work and are grateful for their comments and suggestions. We believe that the proposed revisions have improved the manuscript and made it more complete. In this document, we carefully address all comments and describe the corresponding changes in the revised manuscript. For the readers' convenience, the referee's comments and our statements are color-coded (black: referee comment, blue: author reply). Line references in referee's comments are all with respect to the original manuscript. A marked-up manuscript highlighting all changes using LaTeXdiff is attached to this revised submission.

# Contents

R1 Response to Referee A . . . R1
R2 Response to Referee B . . . R22
R3 Additional Changes . . . R33
R4 Bibliography . . . R34

# R1 Response to Referee A

We would like to sincerely thank the referee for the exceptionally detailed and careful review of our manuscript. The breadth and depth of the 37 comments reflect a substantial investment of time and effort, and we greatly appreciate the rigor and constructiveness of the feedback. We have found the review particularly valuable in improving both the clarity and completeness of the manuscript. Below, we address each comment point by point.

**Comment #1:**

The introduction focuses heavily on planetary and space-based applications and dynamic systems, whereas the analysis is restricted to a static dataset. The authors should clarify this relationship or provide a more focused introduction relevant to helicopter-based surveys.

**Reply:**

We thank the referee for this valuable comment. We agree that the original manuscript, which focused exclusively on controlled hover-flight measurements, did not sufficiently support the broader applicability to dynamic and space-based MGRS scenarios emphasized in the introduction. The original motivation for using hover-flight configurations was that they provide a well-constrained validation benchmark, in which the stationary source alignment enable a robust assessment of the forward model and likelihood formulation under minimal uncertainty in the source–detector separation and relative orientation. While this configuration was therefore well suited for validating the core methodology under controlled conditions, we agree that it did not adequately demonstrate the applicability of the framework to operationally realistic dynamic survey scenarios.

To address this concern, we substantially expanded the scope of the revised manuscript by incorporating dynamic single-pass survey measurements with variable source mixtures. In the revised analysis, we extend the inference task to the more operationally realistic survey configuration, in which source mixture, source location, and source activities are inferred jointly from dynamic flight data acquired under continuously evolving measurement conditions. In addition, we generalized the Bayesian inference framework to support this joint inference under fully dynamic source–detector configurations.

We have furthermore expanded the Discussion section to explicitly address both the limitations of the present helicopter-based validation and the broader applicability of the proposed methodology to other MGRS platforms, including spaceborne and planetary gamma-ray spectroscopy systems. With the newly added dynamic survey analysis, we believe that the revised manuscript now provides a substantially stronger empirical foundation for the broader discussion and motivation presented in the Introduction.

**Changes:**

The corresponding results are presented in the newly added Section 2.4 of the revised manuscript. Furthermore, Sections 2.1, 2.2, 3, 4.5, and 4.6 have been revised to incorporate the extended Bayesian inference framework and to discuss the additional results obtained from the dynamic single-pass survey analyses, as well as to explicitly clarify the applicability of the methodology beyond crewed airborne platforms. All modifications are fully traceable in the attached LaTeX`diff` marked-up manuscript.

**Comment #2:**

Line 15: "dynamically varying source–detector geometries" is slightly inaccurate. Typically, the geometries of the source and detector themselves do not vary (unless dealing with dispersed sources); rather, their positions vary relative to each other and the environment.

**Reply:**

We agree with the referee that the original phrasing was imprecise, as the geometries of the source and detector are typically fixed. In mobile gamma-ray spectrometry, it is instead the relative positioning and orientation (attitude) of the detector with respect to the source and surrounding environment that vary due to platform motion. The text has been revised accordingly to reflect this more accurate description.

**Changes:**

The phrase "dynamically varying source-detector geometries" has been replaced with "variations in source-detector separation and relative orientation induced by platform motion".

**Comment #3:**

Line 27: "empirical calibration libraries" is not clearly defined.

**Reply:**

We agree with the referee that the highlighted term was not clearly defined in the manuscript. We have revised the corresponding paragraph to clarify the term "template library".

**Changes:**

The affected paragraph has be revised as follows:

> "On the template side, spectral-template libraries—defined here as collections of reference detector response spectra for specific radionuclide sources and source–detector configurations—may be derived either from empirical calibration measurements or from numerical simulations. Empirically derived libraries are necessarily sparse, being restricted to a limited set of radionuclide sources and geometries, and their extension to broader survey conditions is prohibitively costly and time-consuming, often requiring days of dedicated measurements and extensive source handling [14, 22]."

**Comment #4:**

Line 34: The claim that approximations fail to reproduce platform-dependent scattering/attenuation is contradicted by Ref. 9, which performs similar modeling.

**Reply:**

We agree with the referee that Ref. 9 presents detailed Monte Carlo modeling of both the platform (truck) and the surrounding environment. While the spectral response presented in Fig. 9 in Ref. 9 shows notable systematic deviations (>100 %) between measured and predicted spectra, the origin of these discrepancies may also involve additional factors beyond platform and environmental modeling,

such as detector response effects, including energy resolution, as discussed by the authors in Ref. 9. Furthermore, Ref. 9 employs a comparatively high lower-energy-threshold of 216 keV, which limits sensitivity to the low-energy regime where Compton scattering in the environment and platform structure is most pronounced. In contrast, the analysis presented in this work explicitly includes spectral information down to 50 keV, thereby probing a regime in which scattering- and attenuation-induced distortions are significantly more pronounced and therefore more critical for accurate template modeling. Based on these considerations, we have decided to remove Ref. 9 from the highlighted sentence to avoid potential ambiguity.

### Changes:

We removed Ref. 9 from the cited references in the highlighted sentence and revised the surrounding text to more precisely specify the photon-energy ranges over which platform-modeling effects and the corresponding improvements from high-fidelity simulations are observed:

> "These approximations fail to reproduce key platform-dependent effects such as scattering and attenuation within the platform structure, resulting in persistent, energy-dependent template biases that can exceed 200 % at photon energies below ~100 keV [1, 11, 20]. Recent advances in high-fidelity Monte Carlo modeling have demonstrated that incorporating detailed dynamic mass models into the simulations can substantially reduce template-related discrepancies, in particular at low photon energies ≤200 keV [4, 7]."

### Comment #5:

Line 45 "overdispersion effects that arise from fluctuating experimental conditions": To ensure clarity, the authors should expand on the physical mechanism driving this. It appears the overdispersion stems from environmental fluctuations occurring on timescales different from those the model accounts for, specifically, where a time-varying integrated probability of detection results in non-Poissonian statistical properties.

### Reply:

We thank the reviewer for this helpful clarification, which allows us to more precisely describe the physical origin of the observed overdispersion.

The key point is that in mobile gamma-ray spectrometry (MGRS), the detected count rate is not strictly stationary over the acquisition interval. Instead, the underlying photon intensity $\lambda(t)$ at the spectrometer location varies due to changes in measurement conditions such as altitude, attitude, ground clearance, background radiation fields, and instrumental or environmental drifts, even under nominally constant source–detector configurations (e.g. hover flights).

When such temporal variability is not explicitly resolved in the inversion model, the recorded counts correspond to an integration over a non-stationary Poisson process rather than a single stationary process. This leads to variance inflation relative to the Poisson assumption, since fluctuations in $\lambda(t)$ contribute additional variability beyond counting statistics alone.

From a statistical perspective, the variability of the observed counts can be decomposed using the law of total variance:

$$\mathrm{Var}(N) = \mathbb{E}[\mathrm{Var}(N \mid \lambda)] + \mathrm{Var}(\mathbb{E}[N \mid \lambda]),$$

where $N$ denotes the total number of detected counts recorded within a given acquisition interval, and $\lambda$ represents the (potentially time-varying) underlying detection rate. The first term corresponds to the intrinsic Poisson counting statistics, while the second term captures additional variability arising from fluctuations in $\lambda$. This decomposition shows that any variability in the detection rate leads to variance inflation beyond the Poisson limit, explaining why $\mathrm{Var}(N) > \mathbb{E}[N]$ is commonly observed in MGRS data under realistic survey conditions.

**Changes:**

Accordingly, we have revised the highlighted part in the introduction as follows:

> "A key limitation of these formulations is the assumption that the observed counts follow a stationary Poisson process. In practice, however, MGRS measurements are subject to continuous fluctuations in background radiation fields, environmental and instrumental response variations, and platform drifts, introducing unmodeled systematics that lead to overdispersion relative to the ideal Poisson model, i.e., larger fluctuations in the observed counts than predicted by the model [9, 17, 28]."

Additional references were included to support these statements.

**Comment #6:**

Line 46: The claim that Poisson-based MLE provides "no rigorous framework for uncertainty quantification" is inaccurate. Rigorous UQ is established in several works (e.g., Ref. 7, 42, `https://doi.org/10.1109/TNS.2020.3024735`, `https://doi.org/10.1109/TNS.2022.3226707`, `https://doi.org/10.48550/arXiv.2412.02926`, `https://doi.org/10.1038/s41598-024-71336-z`.

**Reply:**

We thank the referee for this important comment and for highlighting relevant literature. We agree that Poisson-based maximum likelihood estimation (MLE) can be embedded in a frequentist uncertainty quantification (UQ) framework, for example via Monte Carlo sampling, likelihood-based interval construction, or asymptotic theory.

However, our original statement aimed to highlight practical and methodological limitations that arise specifically in the context of MGRS full-spectrum analysis (FSA). First, rigorous frequentist UQ in high-dimensional inverse problems such as FSA is non-trivial. In particular, the presence of numerous nuisance or hyperparameters (e.g., background components, environmental variability, and likelihood variance parameters) requires either profiling or marginalization. While profiling is commonly used in practice, it is well known to underestimate uncertainties in ill-posed and highly correlated problems, whereas full marginalization is generally not tractable within a purely frequentist MLE framework. This limitation becomes especially pronounced under low-count conditions and strong spectral correlations, where asymptotic approximations break down [13, 30, 31].

Second, we note that most of references shared by the referee focus on simplified or reduced problems, involving monoenergetic analyses or a small number of regions of interest (ROI). In contrast, MGRS FSA involves high-dimensional spectral inversion with strongly overlapping templates and time-varying acquisition conditions. In this setting, the combination of ill-posedness, model mismatch, and high parameter dimensionality significantly complicates the construction of reliable frequentist uncertainty estimates.

**Changes:**

To address these points, we have revised the corresponding part in the introduction to avoid the overly general statement and instead emphasize the practical limitations of standard Poisson-based MLE implementations in the specific context of FSA as follows:

> "In addition, while Poisson-based MLE formulations can, in principle, support uncertainty quantification, their practical application to FSA remains challenging. In particular, the high-dimensional and ill-posed nature of the inversion, the difficulties in handling nuisance parameters, and deviations from ideal Poisson assumptions can lead to unstable solutions and systematically underestimated uncertainties [31, 33]."

with additional references provided to support these statements. In the process of reevaluating the highlighted section, we also noticed that, among the originally cited studies, only Salathe et al. [26] specifically applied the methodology in the context of FSA. Accordingly, we removed the studies [2, 16] from the corresponding paragraph to avoid citing works that are not directly related to FSA-based inversion methodologies.

**Comment #7:**

Section 2.1: This section would benefit from simplified paraphrasing for less experienced readers. For example, defining vector(y)i as gamma-ray histograms, curly d_k as environmental parameters, and theta as the unknowns (activities). The distinction between theta and xi should also be clarified.

**Reply:**

We fully agree with the referee that Section 2.1 was overly technical and insufficiently accessible to less experienced readers. To address this, we have revised both the structure and presentation of the section to improve clarity and pedagogical flow. In addition, we reorganized the methodological presentation by relocating several highly technical derivations and supporting definitions to the dedicated Methods section, retaining in Section 2.1 only the elements required for interpretation of the results. This restructuring also aligns with the broader revision introduced in response to Comment #1, where the methodological framework was extended and consolidated across the manuscript.

**Changes:**

Section 2.1 has been substantially restructured to improve clarity and accessibility. Highly technical derivations and auxiliary definitions have been moved to the Methods section, while the main text has been streamlined to focus on the conceptual structure of the inference framework. In addition, the notation has been clarified by explicitly defining $\mathcal{Y}$ as the set of gamma-ray histograms, $\mathfrak{D}$ as the associated experimental conditions, and $\boldsymbol{\theta}$ as the set of unknown gamma-ray source strengths. The distinction between $\theta$ and $\xi$ has been explicitly clarified throughout the section. Minor textual revisions were also performed to improve readability for non-specialist readers. All modifications are fully traceable in the attached LaTeX`diff` marked-up manuscript.

**Comment #8:**

Line 68: "pulse-height spectral data". It remains unclear if the authors worked in pulse-height (channel) space or energy space; Figure 2 suggests the latter.

**Reply:**

We thank the referee for this important clarification. We agree that the original wording "pulse-height spectral data" did not sufficiently clarify the distinction between pulse-height (channel) space and energy space, which may have led to ambiguity in the interpretation of the results.

To address this, we have revised the manuscript to explicitly clarify that energy-calibrated spectra are used only for visualization in the figures, while the underlying inference is performed in pulse-height (channel) space.

**Changes:**

Clarifying statements have been added in the figure descriptions to indicate that energy-calibrated spectra are shown for visualization purposes only:

> "All spectra shown in energy units are derived from calibrated pulse-height channels for visualization only."

Furthermore, the SAGRS system description has been revised to explicitly describe the detector calibration chain (gain stabilization, linearization, and offset correction) and to clarify that it ensures a stable channel-to-energy relationship, while inference is performed in pulse-height (channel) space as follows:

> "The readout electronics provide automatic gain stabilization by leveraging spectral information from natural potassium and thorium gamma-ray emission lines together with crystal temperature measurements from auxiliary thermocouples. In addition, the system performs spectral linearization with offset correction and live-time tracking. These procedures ensure a stable and approximately linear mapping between pulse-height channels and energy over the duration of a measurement campaign. Consequently, all Bayesian inference is performed in pulse-height channel space, which provides a stable and detector-native representation of the measured spectra."

**Comment #9:**

Line 72: "single point solution" is potentially confusing; "point estimate and confidence interval" are the standard Frequentist terms and both are available in a Frequentist framework.

**Reply:**

We thank the referee for this comment. We agree that the term "single point solution" may be ambiguous in this context and does not reflect standard statistical terminology. In the revised manuscript, we have therefore removed this phrasing to avoid potential confusion and to align the wording with more conventional statistical language.

We further acknowledge the referee's broader point regarding the relationship between Bayesian and frequentist interpretations of point estimates and uncertainty quantification, which we have also addressed in response to comment #6.

**Changes:**

The phrase "single point solution" has been removed and replaced with more standard statistical terminology where applicable. All modifications are fully traceable in the attached LaTeX`diff` marked-up manuscript.

**Comment #10:**

Line 75 equation: The y-subscript for "N in Natural Numbers" is missing. While, consistent, the use of N_curly(y) for both the curly Y definition but also the curly D definition is a bit confusing. Define it first as the number of measurement configurations.

**Reply:**

We thank the referee for this careful observation. We agree that the original manuscript contained a typographical error in the definition of the spectral dimensionality, where the subscript $y$ in $N_y$ was missing. This has been corrected in the revised manuscript.

In addition, we have clarified and generalized the notation. The number of observed spectra is now explicitly denoted by $N_{\mathcal{Y}}$, while the number of experimental condition vectors is independently defined as $N_{\mathfrak{D}}$. This distinction reflects the fact that spectra and experimental conditions are not necessarily in a strict one-to-one correspondence in the most general formulation of the inference problem.

**Changes:**

We have revised the corresponding definitions in Section 2.1 to make this structure explicit and to avoid ambiguity between measurement configurations and auxiliary experimental descriptors. All modifications are fully traceable in the attached LaTeX`diff` marked-up manuscript.

**Comment #11:**

Line 87: "nuisance gamma-ray background". The term "nuisance" is unusual here when referring to cosmic and secondary effects. It is not a nuisance parameter in the Bayesian sense either.

**Reply:**

We thank the referee for this remark. We agree that the term "nuisance gamma-ray background" was misleading in this context, as it does not refer to Bayesian nuisance parameters but to explicitly known background components within the forward model. We have therefore replaced this term throughout the manuscript with "known gamma-ray background components" to avoid ambiguity. For clarity, we note that the corresponding text was also moved from Section 2.1 to Section 4.5 as part of the structural revision carried out in response to Comment #7.

**Changes:**

The term "nuisance gamma-ray background" has been replaced by "known gamma-ray background components".

**Comment #12:**

Line 106: "multiscale". The authors should clarify what is meant by "multiscale" in this context.

**Reply:**

We agree that the term "multiscale" was insufficiently explained in the original manuscript. In the revised version, we clarify that the term refers to the sequence of coupled detector-response processes spanning multiple spatial and temporal scales, ranging from microscopic energy deposition and charge-carrier thermalization to scintillation-light production and subsequent signal formation and processing.

**Changes:**

The corresponding sentence has been revised as follows:

> "... and the energy-conversion chain in the detector system, spanning processes across multiple spatial and temporal scales, including microscopic energy deposition, charge-carrier thermalization, scintillation-light production, and subsequent signal formation and processing [3, 32]."

Furthermore, we have included additional references to provide further support and context for these processes.

**Comment #13:**

Section 2.2: The gain of NaI(Tl) detectors is temperature-sensitive. The authors should note how gain stabilization was maintained (e.g., environmental control in the helicopter bay).

**Reply:**

We thank the referee for this important comment. The SAGRS detector assembly is embedded in thermally insulating and vibration-damping polyethylene foam, as already described in the original manuscript in Section 4.4. In addition, the system continuously records crystal temperatures using auxiliary thermocouples, which are used together with spectral information from natural potassium and thorium emission lines for automatic online gain stabilization and correction. To make this more explicit, we expanded the corresponding system description in the revised manuscript. We also note that a comprehensive technical description and validation of the SAGRS calibration and stabilization pipeline is provided in [4], which is already cross-referenced in Section 4.4.

**Changes:**

The following sentence was added to Section 4.4:

> "The readout electronics provide automatic gain stabilization by leveraging spectral information from natural potassium and thorium gamma-ray emission lines together with crystal temperature measurements from auxiliary thermocouples."

**Comment #14:**

Line 130-136: "RLLSpec". I am unclear if RLLSpec was used for data normalization, as this would violate the count-space requirements of the framework. Also the missing discussion of energy calibration makes me believe the authors mean RLLCal?

**Reply:**

We thank the referee for this important clarification. We agree that the use of the pipeline name `RLLSpec` without further specification may have suggested that all derived data products (including count-rate and net spectra) were used in the inference. We clarify that `RLLSpec` comprises several processing stages, including the extraction of pulse-height count spectra as well as the computation of derived products such as gross and net count-rate spectra. However, only the pulse-height count

spectra are used as input to the Bayesian inference framework in this work. The additional derived quantities are not used in the present analysis. The complementary pipeline `RLLCal` provides the calibration models required for system calibration and energy mapping. All inference is carried out strictly in pulse-height count space, as described in Sections 2.1 and 4.5 as well as discussed in the response to Comment #8. We have revised the manuscript to make this distinction explicit and to avoid any potential confusion.

**Changes:**

The corresponding sentence has been revised to more precisely reflect the actual data products used:

> "Data reduction to retrieve pulse-height count spectra and associated calibration models (energy & spectral resolution) was performed independently for each experiment using the post-processing pipelines `RLLSpec` and `RLLCal` [4]."

In addition, to explicitly link the calibration models to the spectral forward model, we revised the relevant sentence in Section 4.2 and added a cross-reference to Section 2.2:

> "Energy deposition events were recorded per crystal using custom `usreou` routines and converted into expected pulse-height spectra using the `NPScinMC` pipeline, leveraging energy and resolution calibration models as detailed in Ref. [4] (see also Sec. 2.2)."

**Comment #15:**

Line 157: "double-differential flux banks" is an abstract term and requires a brief definition.

**Reply:**

We agree with the referee that the term "double-differential flux banks" was overly abstract and not sufficiently accessible to the reader. In the revised manuscript, and in line with the general simplification of the presentation (cf. Comment #7), we have therefore replaced this terminology with "gamma-ray flux libraries", as the double-differential character is not essential for understanding the results at this stage of the exposition. This change also aligns the terminology with common usage in applied physics, where "library" is preferred over "bank," while the latter is more typical in astrophysics and cosmology. The corresponding formal definitions remain unchanged and are provided in the Methods section (Section 4.1), with appropriate cross-references from Section 2.1 as in the original structure.

**Changes:**

The term "double-differential flux banks" has been replaced by "gamma-ray flux libraries" throughout the manuscript. The underlying formal definition of the flux representation remains unchanged and is provided in Section 4.1, with appropriate cross-references retained in Section 2.1.

**Comment #16:**

Line 163: Radon is treated as both a nuisance source and a fit parameter; this distinction should be explicitly noted.

**Reply:**

We agree that the previous wording was ambiguous regarding the role of radon in the model. In the revised manuscript, and in line with the structural changes introduced in response to Comment #7, we have substantially reorganized this part of the text, moving technical details to Sections 4.5 and 4.6, while presenting only the essential elements for interpretation in Sections 2.3 and 2.4.

To clarify the point raised by the referee, the radon-related background contribution is empirically constrained using dedicated background flights conducted over Lake Thun within $10\,\mathrm{km}$ and less than $1\,\mathrm{h}$ of the experiments. However, to account for residual spatiotemporal variability in radon progeny concentrations, we introduce an additional radon correction term $\Delta\xi_{\text{Rn-222}} \in \mathbb{R}$, which is inferred jointly with the other source strengths in the Bayesian framework.

**Changes:**

The corresponding text in Section 2.3 has been revised as follows:

> "Combined spectral contributions of residual intrinsic, cosmic-ray, and atmospheric radon progeny backgrounds were estimated empirically from independent background flights over Lake Thun at orthometric heights matched to the hover measurements (see Supplementary Fig. S1). Because these flights were conducted within $10\,\mathrm{km}$ and less than $1\,\mathrm{h}$ of the experiments, we considered them representative of the intrinsic and cosmic background at the training site. To account for spatiotemporal variability in radon progeny concentrations, we included an additional variable radon source term $\Delta\xi_{\text{Rn-222}} \in \mathbb{R}$ and inferred it jointly with the other source strengths in the Bayesian analysis (see Section 4.3)."

**Comment #17:**

Line 172: It would be prudent to mention here that the probabilistic model used is a Negative Binomial distribution.

**Reply:**

We agree with the referee that the use of a negative binomial likelihood was not sufficiently explicit in the main text of the original manuscript. In the revised version, we now state this assumption explicitly in Section 2.1. The previously highlighted sentence has been removed in the course of the structural revision carried out in response to Comment #1.

**Changes:**

The following text has been newly added in Section 2.1:

> "Two critical components of this framework are the likelihood function and prior specifications, which together must faithfully reflect the statistical character of MGRS count data and existing domain knowledge. Drawing on treatments in cosmology, nuclear and particle physics [6, 12, 15, 18, 24, 25, 29], we adopt a negative binomial likelihood to generalize the stationary Poisson assumption in prior FSA formulations to the nonstationary count processes characteristic of MGRS, complemented by weakly informative priors to avoid overly restrictive assumptions on the model parameters. The complete likelihood formulation and prior specifications are detailed in Section 4.5."

**Comment #18:**

Section 2.4: While the exact parameters for the prior are provided in the supplementary materials, the choice of prior should be mentioned here and the reason for this choice outlined. The Prior is shown in Fig. 2, but there is no mention of it anywhere in the main text.

**Reply:**

We thank the referee for this important suggestion. We agree that the original manuscript did not sufficiently motivate or describe the choice of prior distributions in the main text. To address this, we have revised the highlighted part in the manuscript to explicitly introduce the prior structure and to clarify the rationale behind the chosen distributions. We also note that the corresponding material has been moved from Section 2.4 to Section 4.5 in response to Comment #7.

**Changes:**

The following paragraph has been added to Section 4.5:

> "To avoid overly restrictive assumptions on these prior distributions, we adopted weakly informative, statistically independent marginal priors $\pi(\theta \mid \mathcal{S}) := \prod_{i=1}^{M} \pi\left(\theta_i \mid \mathcal{S}\right)$ for all $M$ model parameters $\theta$. For parameters that are constrained to half-spaces, in particular the source strengths and the dispersion parameter, we adopt truncated univariate normal distributions with support restricted to the physically admissible domain. For parameters defined on finite bounded domains, such as the source positions in single-pass survey configurations, we employ uniform priors, reflecting the absence of prior spatial preference within the measurement area. A full list of all marginal prior distributions is provided in Supplementary Table S2."

We also note that in response to Comment #21 by Referee B, we have removed the shaded prior visualization in Fig. 2 to improve accessibility of the figure, in particular for readers with color vision deficiencies.

**Comment #19:**

Figure 2: The "Prior" (grey) is difficult to see and should be darkened. The K-40 line appears wider on the right; the authors should clarify if this is a plotting artifact. I would strongly suggest including residual plots (deviance or squared) to support the claim of model alignment.

**Reply:**

We thank the referee for this helpful comment. In response to Comment #21 by Referee B, we have revised Fig. 2 to improve overall readability. In particular, the simultaneous display of two semi-transparent components (prior and posterior) was found to reduce visual clarity, especially for readers with color vision deficiencies. We therefore removed the prior shading to improve accessibility of the figure, while ensuring that all prior information remains fully specified and accessible through the formal model description (see also Comment #18).

We further agree that the inclusion of residual diagnostics is important for assessing model adequacy. We have therefore added standardized residuals for all spectra in Figs. 2, 4, 5, and 6 to support quantitative evaluation of model-data agreement.

Regarding the apparent asymmetry of the K-40 line, we clarify that the same spectral resolution model was used for the K-40 templates used in both panels. We confirm that the difference in apparent peak shape is purely a visualization effect arising from binning and axis scaling, not a physical difference. The K-40 templates provide a reference showing that both cases are consistent, which is also confirmed by the residuals.

**Changes:**

Fig. 2 has been updated by removing the prior shading to improve visual clarity and accessibility, in particular for readers with colour vision deficiencies. In addition, standardized residuals have been added to Figs. 2, 4, 5, and 6 to provide a quantitative assessment of model agreement across all presented cases.

**Comment #20:**

Line 202 - 206: The interpretation of elevated overdispersion at longer sampling times feels like conjecture. It is more likely that the model is simply not accurate enough to account for second-order effects (e.g., sky-shine, back-scattering, or mass model inaccuracies) at 5 min integration time. If the authors believe their model is sufficiently accurate to describe the data at that level of fidelity they should provide proof or references.

**Reply:**

We thank the referee for this careful comment. We agree that the original wording may have over-interpreted the observed dependence of the dispersion parameter and have therefore moderated the language throughout this section. We also note that the forward model already accounts for dominant second-order environmental effects, including atmospheric contributions (modeled up to a radius of $3\,\mathrm{km}$) as well as ground reflections and back-scattering from soil and infrastructure, including paved surfaces (see Section 4.3). Furthermore, detailed validation of the underlying Monte Carlo model was performed in [4], demonstrating good agreement with experimental measurements across representative source–detector configurations and environmental conditions for extended acquisition times ($\geq 5\,\mathrm{min}$). Nevertheless, as correctly pointed out by the referee, residual unmodeled effects and/or slow temporal variations cannot be fully excluded and may contribute to the observed behavior.

**Changes:**

The description of the dispersion behavior has been revised to avoid causal interpretation and to present the result in a more conservative manner:

> "In contrast, the dispersion parameter $\alpha_{\mathrm{NB}}$ exhibits a systematic dependence on acquisition duration, with larger values for the full-length datasets compared to short integration times, corresponding to a statistically significant separation between both regimes at the $>8\sigma$ level. This behavior indicates mild overdispersion relative to the ideal Poisson counting model, which becomes more pronounced for longer integration times. While the forward model accounts for dominant second-order environmental effects, including atmospheric contributions and ground back-scattering (see Section 4.3), residual unmodeled systematics may still contribute to the observed trend. Importantly, this behavior emerges despite the use of a single forward model and spectral template across all acquisition times for a given source configuration, suggesting that the observed increase in dispersion is unlikely to be

> explained by a static model mismatch alone. It may instead reflect measurement-condition-dependent variability at longer integration times, although contributions from higher-order physical effects (e.g. residual environmental scattering or subtle model inaccuracies) cannot be fully excluded."

**Comment #21:**

Line 221: Accuracy is reported by comparing posterior medians to reference activities. The authors should clarify if these "reference values" were measured or derived from data sheets.

**Reply:**

We thank the referee for this important clarification request. The reference activities used in this study are fully documented in Section 2.2. In particular, the activities of the anthropogenic sources ($^{137}_{55}$Cs and $^{133}_{56}$Ba) are based on laboratory gamma-ray assays performed and certified by the source manufacturer. In contrast, natural background activity concentrations of terrestrial potassium $K_{nat}$, uranium $U_{nat}$ (with progeny), and thorium $Th_{nat}$ (with progeny) were constrained by *in-situ* gamma-ray spectroscopic measurements at 23 reference locations across the training site [10]. We have clarified this distinction in the revised manuscript to avoid ambiguity between manufacturer-certified source activities and field-derived background estimates.

**Changes:**

A cross-reference to Section 2.2 has been added:

> "We assess the accuracy of the inferred anthropogenic source activities for $^{137}_{55}$Cs and $^{133}_{56}$Ba by comparing the posterior median activities with independently determined reference activities (see Section 2.2)."

The corresponding part in Section 2.2 has also been revised for clarity as follows:

> "Reference activities of the anthropogenic sources were obtained from manufacturer-certified calibration certificates based on laboratory gamma-ray assays. Natural background activity concentrations of terrestrial potassium $K_{nat}$, uranium $U_{nat}$ (with progeny), and thorium $Th_{nat}$ (with progeny) were constrained by *in-situ* gamma-ray spectroscopic measurements at 23 reference locations across the training ground [10]."

**Comment #22:**

Line 229-230: While radon seems stable, alpha certainly varies in width and location.

**Reply:**

We agree with the referee that this statement was not sufficiently precise. In the context of the structural revisions introduced in response to Comment #1, the corresponding passage has been removed from the manuscript.

**Changes:**

The referenced statement has been removed.

**Comment #23:**

Line 230 - 232: This phrase is unclear. The authors talk about intrinsic limits, I believe the limits are the models accuracies, not the method.

**Reply:**

We agree with the referee that the phrasing was unclear and potentially misleading regarding the origin of the observed limitations. In the context of the structural revisions introduced in response to Comment #1, the corresponding passage has been removed from the manuscript. A more detailed discussion of potential model-related inaccuracies and their implications is provided in the revised response to Comment #20.

**Changes:**

The referenced passage has been removed.

**Comment #24:**

Line 239 - 240: "statistically insignificant" deviations. As these sit near the 68% credible interval, they are not as credible as this sounds, which raises the possibility of systematic bias and missing second order effects.

**Reply:**

We thank the referee for this observation and agree that the interpretation of credible intervals requires care. In the Bayesian framework, credible intervals represent probability mass regions and do not correspond to strict acceptance or rejection thresholds. Consequently, values near the boundary of the 68% credible interval remain fully consistent with the posterior distribution and do not, by themselves, indicate systematic bias. In applied physics, evidence for systematic discrepancies typically requires deviations at or above the $3\sigma$-level, which is beyond the range of the observed differences in this study.

We further recognize that assessing compatibility also requires accounting for the uncertainty of the independently determined reference activities. To address this point, we have added $1\sigma$ uncertainty bounds corresponding to the certified laboratory assay uncertainties provided by the source manufacturer in Fig. 3. As evident from the revised figure, the inferred posterior distributions remain fully compatible with the independently determined reference values within the combined uncertainty budget.

**Changes:**

The manuscript text has been clarified to improve the interpretation of of the statistical significance of the observed deviations as follows:

> "Importantly, all deviations remain statistically insignificant within the combined $1\sigma$ uncertainties of the posterior estimates and the reference activities."

In addition, Fig. 3 has been updated to include $1\sigma$ uncertainty bounds on the independently determined reference activities for the anthropogenic radionuclides provided by the source manufacturer.

**Comment #25:**

Line 247: The authors mention reference values for terrestrial components. What are these reference values? If there are ground truth data, they should be also added to Fig 2.

**Reply:**

We thank the referee for this question and clarify that the "reference values" for terrestrial components refer to independent *in-situ* constraints. Specifically, natural background activity concentrations of $K_{nat}$, $U_{nat}$ (with progeny), and $Th_{nat}$ (with progeny) were derived from dedicated in-situ gamma-ray spectroscopic measurements at 23 reference locations across the training site [10]. These values provide broad empirical constraints rather than point-like reference standards.

We have clarified this distinction in the revised manuscript to avoid ambiguity between manufacturer-certified anthropogenic source activities and field-constrained background estimates. Given the relatively large dispersion of these background components (see gray regions in Fig. 3), we did not introduce additional shaded "reference" overlays in Fig. 2 to avoid visual clutter, in line with the figure simplification adopted in response to Comment #21 of referee B.

**Changes:**

The description of reference values for natural terrestrial background components has been clarified to avoid ambiguity with manufacturer-certified anthropogenic source activities. In particular, we now explicitly state that these values correspond to *in-situ* constraints. The relevant sentence in Section 2.2 has been revised as follows:

> "Natural background activity concentrations of terrestrial potassium $K_{nat}$, uranium $U_{nat}$ (with progeny), and thorium $Th_{nat}$ (with progeny) were constrained by *in-situ* gamma-ray spectroscopic measurements at 23 reference locations across the training ground [10]."

In addition, the term "reference values" has been replaced by "*in-situ* constraints" in Section 2.3 to avoid ambiguity. The corresponding sentence now reads:

> "Nevertheless, for all experiments, the posterior activity estimates are statistically consistent with the corresponding *in-situ* constraints as well as with each other within their combined uncertainty ranges."

**Comment #26:**

The authors should discuss the functional abilities and limits of the model in more detail: Can it detect the absence or presence of a source, identify isotopic composition of sources, detect multipe sources at once, or quantify distributed sources?

**Reply:**

We thank the referee for this insightful suggestion. As discussed in response to Comment #1, we agree that the original manuscript was too narrowly framed around static hover-flight measurements and did not sufficiently articulate the functional scope of the proposed framework. In the revised manuscript, we therefore clarify and expand the methodological scope to explicitly address the functional capabilities

of the model. In particular, the framework now supports joint inference of source mixture, activity, and location under both static and dynamic survey conditions. This includes the ability to resolve multiple simultaneous sources and to infer heterogeneous source mixtures within the limits of the available spectral and spatial information. We have further extended the analysis to dynamic single-pass survey data, which allows us to demonstrate the performance of the method under continuously varying source–detector geometries. In addition, the Discussion has been expanded to clarify both the demonstrated capabilities and the current limitations of the approach, in the context of broader MGRS applications.

### Changes:

Sections 2.1, 2.2, 3, 4.5, and 4.6 have been revised to incorporate the extended Bayesian inference framework and to include additional results from the dynamic single-pass survey analyses, as well as to explicitly clarify the applicability of the methodology and its limitations. All modifications are fully traceable in the attached LaTeX`diff` marked-up manuscript

### Comment #27:

Line 252 - 253: The authors claim to prove the framework for "MGRS," but the study is technically limited to static GRS.

### Reply:

We thank the referee for this comment. As discussed in response to Comment #1, we agree with the referee that the original formulation was too limited by restricting the analysis to static hover-flight measurements. In the revised manuscript, we have extended both the analysis and the discussion to explicitly include dynamic single-pass survey data, thereby demonstrating the applicability of the framework to general MGRS configurations beyond static GRS.

### Changes:

The manuscript has been revised to clarify the scope and applicability of the framework. Specifically, dynamic single-pass survey data have been added to the analysis, and Sections 2.1, 2.2, 2.4, 3, 4.5, and 4.6 have been updated accordingly. All changes are fully traceable in the attached LaTeX`diff` marked-up manuscript.

### Comment #28:

Line 255: This statement is too vague. Many publications correctly handle sparse counting statistics (albit in a Frequentist framework). It is also unclear to me what improvements the authors did to with respect to spectral template modeling.

### Reply:

We thank the referee for this important comment. We agree that the original formulation was too vague and did not sufficiently emphasize the key methodological advances of the present work, in particular with respect to existing sparse-counting approaches and spectral template-based FSA methods. We would like to clarify that the main contribution of this work is not the introduction of sparse counting statistics or spectral template modeling, which are indeed well established in both frequentist and Bayesian contexts. Instead, the central advances of the present study are:

1. We demonstrate that posterior exploration and Bayesian evidence estimation become computationally tractable for MGRS inverse problems through a two-stage forward-modeling strategy in which high-fidelity radiation transport is decoupled from inference. Instrument response functions and gamma-ray flux libraries are precomputed on HPC infrastructure, while survey-specific spectral responses are assembled rapidly during inference. This reduces the cost of likelihood evaluations by several orders of magnitude, enabling, for the first time, full-spectrum Bayesian inference at operational scale while retaining the fidelity of the underlying radiation-transport model across the full energy range relevant to MGRS.

2. We show that source mixtures, source locations, and source activities can be inferred simultaneously from full-spectrum MGRS data within a single probabilistic framework. In contrast to conventional FSA pipelines, which treat these components separately or sequentially, often addressing only a subset of the full inference problem, the proposed approach enables fully coupled inference under conditions of low-count statistics and strong spectral overlap. Quantitatively, this results in percent-level agreement in reconstructed source activities relative to independent laboratory reference values, corresponding to an order-of-magnitude improvement over previously reported FSA-based approaches under comparable conditions. In addition, the framework achieves robust source mixture identification with decisive statistical evidence, exceeding $>5\sigma$ statistical significance even in challenging multi-source scenarios where weak radionuclide signatures are strongly obscured by dominant sources.

**Changes:**

The Discussion section has been substantially rewritten to explicitly clarify the methodological contributions and to better distinguish the proposed framework from existing sparse-counting and spectral template-based FSA approaches. All modifications are fully traceable in the attached LaTeX`diff` marked-up manuscript.

**Comment #29:**

Line 260 - 261: "realistic MGRS conditions". A static hover does not represent the background fluctuations and spectral dynamics of a single-pass survey.

**Reply:**

We thank the referee for this comment. We agree that the wording "realistic MGRS conditions" in the original manuscript was insufficiently precise and could be misleading in isolation. As already clarified in our response to Comment #1, the revised manuscript now includes both controlled hover-flight and dynamic single-pass survey configurations, which together cover the relevant operational MGRS conditions. We have revised the text accordingly to improve clarity and avoid ambiguity.

**Changes:**

The wording in the Discussion has been revised to more precisely distinguish between controlled hover-flight validation and dynamic single-pass survey configurations. All changes are fully traceable in the attached LaTeX`diff` marked-up manuscript.

**Comment #30:**

Line 268 - 269: The blanket statement is not characterizing previous work accurately.

**Reply:**

We thank the referee for this comment. We agree that the original wording was overly general in stating that conventional methods "tend to underestimate uncertainty or yield unstable solutions." We have removed the highlighted statement from the Discussion.

**Changes:**

The highlighted statement has been removed from the Discussion to avoid overgeneralized statements. All modifications are fully traceable in the attached LaTeX`diff` marked-up manuscript.

**Comment #31:**

Line 276 - 277: Another blanket statement that is not substantiated. The Possion assumption doesn't bias the source-strength estimate. If biases are observed they are analysis or modeling artifacts.

**Reply:**

We thank the referee for this comment. We agree that the original statement was overly strong in attributing bias or uncertainty effects directly to the Poisson assumption. As correctly pointed out, deviations from expected performance are generally not a consequence of the Poisson model itself, but rather arise from model bias, analysis choices, or additional unmodeled systematics in the forward or inverse problem formulation. We have therefore revised the manuscript and removed the corresponding blanket statement to avoid potential misinterpretation.

**Changes:**

The corresponding statement has been removed from the manuscript. All modifications are fully traceable in the attached LaTeX`diff` marked-up manuscript.

**Comment #32:**

LIne 280 - 281: Substantially stronger deviation from Poisson statistics are only expected if the model is insufficiently accurate to capture the data dynamics. It is not a statistical property.

**Reply:**

We thank the referee for this comment. We respectfully note that the statement in the manuscript does not claim that deviations from Poisson statistics arise as an intrinsic property of the Poisson distribution itself. Rather, the intended meaning is that in operational MGRS measurements the assumption of a stationary Poisson process is generally violated due to inherent variability in the underlying intensity over the acquisition interval.

As already discussed in response to Comment #5, the detected counts in MGRS can be more appropriately described as arising from a non-stationary or mixed Poisson process, where fluctuations in the underlying rate induced by environmental, platform motion, and instrumental variations introduce additional variance beyond the Poisson limit. From this perspective, the observed overdispersion reflects unresolved variability in the measurement process, which is not captured by a strictly stationary Poisson likelihood.

**Changes:**

In response to Comment #28, we have substantially rephrased and repositioned the Discussion section, during which we also decided to remove the highlighted statement from the manuscript. All modifications are fully traceable in the attached LaTeX`diff` marked-up manuscript.

**Comment #33:**

Lines 359 - 361: This implies the simulation of pulse-height spectra in channel space. This should be clearly stated in the Results section, or clarified otherwise here.

**Reply:**

We agree that the original wording was not sufficiently explicit regarding the representation space used for spectral simulation and inference. As clarified in response to Comment #8, inference is performed in pulse-height (channel) space, and energy-calibrated representations are used only for visualization purposes.

**Changes:**

Clarifying statements have been added in the figure descriptions to indicate that energy-calibrated spectra are shown for visualization purposes only:

> "All spectra shown in energy units are derived from calibrated pulse-height channels for visualization only."

Furthermore, we added a clarification statement in Section 4.4:

> "...all Bayesian inference is performed in pulse-height channel space, which provides a stable and detector-native representation of the measured spectra."

**Comment #34:**

Line 368: "2×109 primaries distributed across 102 independent runs". Please clarify what is meant by "primaries" and "runs."

**Reply:**

We agree that the terms "primaries" and "independent runs" were not sufficiently defined in the original manuscript. In this context, "primaries" refers to the total number of primary particle histories simulated in the Monte Carlo transport, corresponding in the present study to decays of the predefined radionuclide sources. "Independent runs" refers to statistically independent simulation batches initialized with different random seeds, used to distribute the total number of histories across the available HPC infrastructure and improve statistical sampling.

**Changes:**

A clarifying statement has been added in Section 4.3:

> "The accumulated computation time was $\sim 10^2$ core-hours per source, with $2 \times 10^9$ primary particle histories, where each history corresponds to a full Monte Carlo transport cascade initiated by a single radionuclide decay according to the predefined source emission model, distributed across $10^2$ statistically independent simulation batches initialized with different random seeds for parallel execution and improved statistical sampling on the HPC cluster."

**Comment #35:**

Line 427 - 429: The fact that a Gaussian prior distribution was used should be mentioned here.

**Reply:**

We agree that the original manuscript did not explicitly state the use of a Gaussian prior at this location. We have therefore revised the text to explicitly mention the Gaussian prior distribution used in the model.

**Changes:**

The corresponding part in Section 4.5 has been revised to explicitly state the use of a Gaussian prior distribution for the relevant model parameters:

> "To avoid overly restrictive assumptions on these prior distributions, we adopted weakly informative, statistically independent marginal priors $\pi(\theta \mid \mathcal{S}) := \prod_{i=1}^{N_s+1} \pi(\theta_i \mid \mathcal{S})$ for all model parameters $\theta$. For parameters that are constrained to half-spaces, in particular the source strengths and the dispersion parameter, we adopt truncated univariate normal distributions with support restricted to the physically admissible domain. For parameters defined on finite bounded domains, such as the source positions in single-pass survey configurations, we employ uniform priors, reflecting the absence of prior spatial preference within the measurement area. A full list of all marginal prior distributions is provided in Supplementary Table S2."

**Comment #36:**

The text is well-written and very carefully and accurately phrased. It is very mathematical, in my opinion, to the extent that the authors' true intentions are often hidden behind abstract and overly complex concepts. While the technical rigor is commendable, the high level of formal complexity may present a barrier to accessibility, even for experts in the field. This isn't a criticism of the depth itself, but a greater emphasis on the underlying intuition alongside the formalism would ensure the work's significant contributions are more easily absorbed. Overall, the use of the English language is exceptional.

**Reply:**

We appreciate the positive assessment of the technical rigor and English quality, and we agree that certain parts of the manuscript were written at a level of abstraction that may reduce accessibility for a broader expert readership. To address this, we have revised the manuscript with a stronger emphasis on conceptual clarity and intuition alongside the formal derivations.

**Changes:**

Several sections have been restructured to more clearly separate conceptual explanations from formal derivations, and additional explanatory text has been added to emphasize the physical interpretation of the mathematical framework. In addition, technical and methodological details not strictly required to follow the main results were moved from the Results section to the Methods section to improve accessibility for non-specialist readers and maintain focus on the primary findings. All modifications are fully traceable in the attached LaTeX`diff` marked-up manuscript.

**Comment #37:**

The list of references is extensive and complete. Inclusion of the planetary references only make sense if they are relevant for the Bayesian methodology.

**Reply:**

We agree that the inclusion of planetary and space-based references must be clearly justified within the scope of the manuscript. As already discussed in response to Comment #1, these references are included to emphasize the general applicability of the proposed Bayesian full-spectrum inference framework beyond the specific crewed airborne validation scenario considered here.

**Changes:**

No changes to the reference list were required. The Introduction and Discussion sections have been revised (see response to Comment #1) to clarify and discuss the relevance of planetary and space-based MGRS applications for the proposed Bayesian methodology. All modifications are fully traceable in the attached LaTeX`diff` marked-up manuscript.

## R2 Response to Referee B

We would like to thank the referee for the detailed feedback as well as for the suggestions on how to improve the manuscript. Below we address all the concerns and questions raised by the referee.

**Comment #1:**

There is one issue with the way it is presented which is easily dealt with. The authors have concentrated on characterizing the energy spectrum and emission strengths of the radioisotope sources. These sources have other characteristics though; they have a location, they may have a shape and an extent, and this work does not present a quantification of these other attributes. Throughout, but especially in the abstract and introduction, the authors should be careful to specify when they talk about 'quantifying the source' that it is the radioisotope source strength, or the energy spectrum, that they are quantifying and it is not a full source-characterization quantification.

**Reply:**

We fully agree with the referee that the original manuscript did not always clearly distinguish between quantification of source activities and radionuclide composition on the one hand, and complete source characterization on the other.

The methodology presented in this revised manuscript is designed to infer source mixtures, source activities, and source locations from MGRS data, but does not attempt to reconstruct all possible source attributes, such as source geometry, spatial extent, or shape. We therefore agree that statements referring to "source quantification" could be interpreted too broadly. We kindly refer to our response to Comment #1 by referee A for a detailed justification and motivation for the broadened scope of the revised manuscript.

To address the referee's concern, we carefully revised the manuscript to ensure that the scope of the inferred quantities is stated explicitly throughout. In particular, we replaced several generic references to "source quantification" with more precise formulations specifying source mixtures, source activities, and source locations. We also reviewed the Abstract, Introduction, Results, and Discussion sections to ensure that the capabilities and limitations of the proposed framework are described consistently and without overstating the degree of source characterization achieved.

**Changes:**

The corresponding statement in the Abstract has been revised as follows:

> "Here, we show that integrating high-fidelity, platform-dynamic Monte Carlo simulations and Bayesian inference with mobile gamma-ray spectrometry enables rapid and accurate inference of the source mixture, associated source activities, and source locations for both distributed and point-like gamma-ray sources."

In addition, we have revised the terminology throughout the manuscript to explicitly distinguish the inference of source mixtures, source activities, and source locations from complete source characterization. All modifications are fully traceable in the attached LaTeX`diff` marked-up manuscript.

**Comment #2:**

[Abstract] What are you quantifying? Location? Emission rate? Dose rate? Radioisotope concentration? This should be clarified.

**Reply:**

We thank the referee for this comment. We agree that the quantities inferred by the proposed framework should be stated explicitly. This concern is closely related to Comment #1, and has been addressed by revising the Abstract and related sections of the manuscript to clearly distinguish between inference of source mixtures, source activities, and source locations, and complete source characterization.

**Changes:**

Please see the response to Comment #1. The corresponding statement in the Abstract has been revised to explicitly state the inferred quantities. All modifications are fully traceable in the attached LaTeXdiff marked-up manuscript.

**Comment #3:**

[Abstract] Again - what are you quantifying about them, they have many quantifiable characteristics.

**Reply:**

We thank the referee for this comment. This concern is addressed in our response to Comment #1, where we clarified throughout the manuscript that the proposed framework infers source mixtures, source activities, and source locations, rather than performing complete source characterization.

**Changes:**

Please see the response to Comment #1. The corresponding terminology has been revised throughout the manuscript and is fully traceable in the attached LaTeXdiff marked-up manuscript.

**Comment #4:**

[Line 4] suggest 'uncrewed' as more inclusive word

**Reply:**

We thank the referee for this suggestion and agree that "uncrewed" is the more inclusive term.

**Changes:**

The corresponding wording has been adopted throughout the manuscript where applicable.

**Comment #5:**

[Line 9] To the citation for uncrewed mobile survey, suggest add C.M. Chen, L.E. Sinclair, R. Fortin, M. Coyle, C. Samson, In-flight performance of the Advanced Radiation Detector for UAV Operations (ARDUO), Nuclear Instruments and Methods in Physics Research Section A: Accelerators, Spectrometers, Detectors and Associated Equipment, Volume 954, 2020, 161609

Sinclair, L.E., Chen, C.M. Inspection of a Restricted Access Site Using UAV Perimeter Survey with the ARDUO Direction-Capable Gamma Spectrometer. Pure Appl. Geophys. 178, 2779–2788 (2021)

**Reply:**

We thank the referee for providing the relevant literature suggestions.

**Changes:**

The suggested references have been added to the manuscript to further support the discussion of uncrewed MGRS applications.

**Comment #6:**

[Line 51] here as well - quantifying emission rate - emission spectrum - radioisotope concentration - spatial distribution?

**Reply:**

We thank the referee for this important clarification request. We have revised the text to explicitly state what is being quantified throughout the manuscript, namely source mixture, associated activities, and source locations.

**Changes:**

Please see the response to Comment #1. The corresponding terminology has been revised throughout the manuscript and is fully traceable in the attached LaTeX`diff` marked-up manuscript.

**Comment #7:**

[Line 56] Finally! It is source strength you are quantifying - good, we need that in the abstract and everywhere in the into where you just talk about 'quantifying the source'

**Reply:**

We thank the referee for this clarification. We refer to our response to Comment #1, where we explicitly clarify how the manuscript has been revised to specify the inferred quantities, as well as to Comment #1 by Referee A, which motivates the broadening of the scope of the work from source activity estimation to joint inference of source mixtures, source activities, and source locations.

**Changes:**

The term "quantifying the source" has been replaced throughout the manuscript with more precise wording referring to source mixtures, source activities, and source locations (see also responses to Comment #1 by Referee A and Comment #1 by Referee B).

**Comment #8:**

[Line 82–83] Do you mean a specific radioisotope - or a specific location as well? Clarify text

**Reply:**

We thank the referee for this clarification request. In response to Comment #1 by Referee A, we have substantially revised the manuscript to clarify how the spectral templates depend on the full set of experimental conditions. In particular, we now explicitly distinguish between cases where the source position is treated as known (e.g. in the hover-flight validation experiments) and cases where it is inferred jointly with the source parameters (e.g. the dynamic single-pass surveys). In the latter case, the source position is included in the parameter vector $\theta$ and inferred within the Bayesian framework, whereas in the former it is treated as a fixed conditioning variable of the forward model. This distinction has been made explicit in Sections 4.1 and 4.5, where the formal definition of the model hierarchy is provided.

**Changes:**

The corresponding text has been revised and partially relocated to Sections 4.1 and 4.5 to ensure a consistent and explicit distinction between fixed experimental conditions and inferred parameters. The role of source position within the model has been clarified in the context of dynamic survey scenarios, while hover-flight cases are now explicitly defined as conditional forward-model evaluations with known geometry. All modifications are fully traceable in the attached LaTeX`diff` marked-up manuscript.

**Comment #9:**

[Figure 1 (a)] Why are these distances circling the source and spectrum - as if its a cycle and once the distance goes past 1 km it's going to cycle back to 0 m? I like the pictograms and the altitude information but graphic should not imply a cycle encircling the sources - suggest a straight vertical line

**Reply:**

We agree with the referee that the original graphic could be misleading in suggesting a cyclic interpretation of the distance annotation. This was an unintended visual artifact of the layout choice. We have revised the figure accordingly to use a linear vertical representation.

**Changes:**

Figure 1(a) has been updated to replace the circular distance representation with a straight vertical arrow to avoid any implication of periodicity.

**Comment #10:**

[Figure 1 (b) & (c)] This is quite nice

**Reply:**

We thank the referee for this positive remark.

**Changes:**

No changes were made.

**Comment #11:**

[Figure 1 (d)] d seems at first to be just the 'gamma ray transport' and 'mobile platform' pictures. Consider some kind of box to show that sub-figure 'd' extends all the way to the pulse-height spectrum. I think there is something wrong with the headings in sub-figure 'd' because 'gamma-ray transport' is continuing to and within the detector medium whereas it seems to be only indicated between emission and the platform

**Reply:**

We thank the referee for this helpful suggestion and agree with the clarification.

**Changes:**

We have replaced the term "gamma-ray transport" with "gamma-ray sources" and slightly rearranged the elements of Figure 1(d) to avoid ambiguity and to clearly indicate that the schematic extends through to the pulse-height spectrum, including the detector response.

**Comment #12:**

[Line 94] Consider improved language here - an 'accurate approximation'? Would that be a 'numerical approximation with quantifiable precision and accuracy'?

**Reply:**

We thank the referee for this helpful suggestion and agree that the original wording was imprecise. We have revised the sentence accordingly to improve clarity.

**Changes:**

The corresponding sentence has been rephrased as follows:

> "While both the posterior and Bayesian evidence are analytically intractable for general likelihood and prior distributions [13], advances in Bayesian computation over the past decades have enabled their numerical estimation with controllable accuracy and precision."

**Comment #13:**

[Line 97–98] the required accuracy and precision determine the number of evaluations which is adequate - can you comment about that

**Reply:**

We thank the referee for this important comment. The required number of likelihood evaluations, as the referee correctly stated, is not fixed a priori, but is determined by the desired level of convergence and statistical precision of the posterior and evidence estimates, as well as the complexity and dimensionality of the inference problem. We have clarified this point in the revised manuscript and explicitly relate the chosen sampling depth to standard convergence diagnostics and effective sample size criteria.

**Changes:**

The corresponding sentence has been revised as follows:

> "However, these methods remain computationally demanding, often requiring $\mathcal{O}(10^4)$ likelihood evaluations for a single inference task in MGRS-like inverse problems, depending on convergence and precision requirements (see Sec. 4.6)."

In addition, Section 4.6 now explicitly details how convergence and sampling adequacy were assessed:

> "The convergence and precision of the MCMC simulations were carefully assessed using standard diagnostic tools [8, 13], showing a potential scale reduction factor $\hat{R} < 1.02$ and effective sample size ESS $> 600$ across all MCMC runs. Additional trace and convergence plots for the individual parameters $\theta$ and point estimators (Supplementary Figs. S2–S6) alongside detailed Bayesian inversion summaries (Supplementary Tables S3 and S4) can be found in the Supplementary Information File for this study."

**Comment #14:**

[Line 113–117] I think this approach is quite related to something the Nevada guys (Piotr et al.) do in their in-house data analysis software. - at least the aspect of pre-computing the instrument response functions and then using those for different experimental scenarios, altitudes etc. They don't publish a lot - but you know them, you could reach over and see if they have anything public you could cite to make the connection.

**Reply:**

If the referee is referring to the AVID software, this appears—after confirmation with Dr. Piotr Wasiolek at the 2024 Technical Meeting in Las Vegas—to be based on a peak-fitting and spectral window approach rather than the full-spectrum modeling framework presented in this study. Furthermore, to our knowledge, this software has not been described in any peer-reviewed publication. We acknowledge the work of the group highlighted by the referee through Ref. [21] in the revised manuscript.

**Changes:**

No changes required.

**Comment #15:**

[Line 132] precision needed - were the sources individually aligned with the yaw axis? was the line between the two sources aligned with the yaw axis?

**Reply:**

We agree with the referee that the original wording was ambiguous regarding the source alignment. The description has been clarified in the revised manuscript to explicitly state the geometric reference used for alignment.

**Changes:**

The sentence has been revised to clarify the alignment relative to the aircraft symmetry axis.

> "In the first configuration, the SAGRS system operated in a static hover-flight mode for $\sim 5\,\mathrm{min}$, with the helicopter positioned at a ground clearance of $\sim 90\,\mathrm{m}$, such that the individually deployed radionuclide point sources on the ground were aligned with respect to the aircraft vertical symmetry axis."

**Comment #16:**

[Line 135] These symbols need to be defined before they are used. You could do, eg, "... activity concentrations of terrestrial K, U and Th (K_nat, U_nat, Th_nat respectively) ...

**Reply:**

We thank the reviewer for this helpful suggestion. We agree that the symbols should be clearly defined at first use, and this has now been addressed in the revised manuscript.

**Changes:**

We have explicitly defined the notation for the natural background activity concentrations at first occurrence as follows:

> "Natural background activity concentrations of terrestrial potassium $\mathrm{K}_{\mathrm{nat}}$, uranium $\mathrm{U}_{\mathrm{nat}}$ (with progeny), and thorium $\mathrm{Th}_{\mathrm{nat}}$ (with progeny) were constrained by *in-situ* gamma-ray spectroscopic measurements at 23 reference locations across the training ground [10]."

**Comment #17:**

[Line 138–139] In a normal aerial survey, the 5 min of acquisition will integrate over a very large area on the ground and be useful only in rare circumstances - suggest discuss this. How did you choose which 1s and which 5s interval to analyze? Is it the first one, a random one?

**Reply:**

We agree with the referee that a $\sim 5\,\mathrm{min}$ acquisition is longer than typically used in conventional airborne surveys based on inorganic scintillator systems. The inclusion of this acquisition time is not intended to represent a typical operational choice for inorganic scintillator based airborne systems, but rather to extend the analysis across a broader range of integration times relevant to MGRS applications.

To ensure that such a comparison is physically meaningful, the static hover-flight configuration provides a controlled and repeatable source–detector geometry. As discussed in Section 2.3 in the main study, this controlled setting is particularly suitable for studying general statistical behaviour relevant to MGRS systems operating across different regimes, including lower-sensitivity airborne HPGe-based systems [34] and spaceborne gamma-ray spectrometry missions [19, 23], where longer effective integration times are common.

The $1\,s$ and $5\,s$ spectra were selected using random starting points within the measurement interval. The $5\,s$ spectra were constructed from five consecutive $1\,s$ acquisitions to ensure consistency between temporal resolutions.

We further note that all dynamic survey results presented in Section 2.4 use a $1\,Hz$ acquisition rate representative of operational airborne surveys.

**Changes:**

We have added a clarifying remark and additional references to motivate the selected acquisition times:

> "We present the results from these Bayesian computations as posterior predictive distributions in Fig. 2 alongside univariate and bivariate marginal posterior distributions shown in Fig. 3 for acquisition times of $1\,s$, $5\,s$, and the full measurement duration ($\sim 5\,min$), spanning the range commonly encountered in MGRS applications [21, 23, 27, 34]."

In addition, as already discussed in response to Comments #1 and #28 by referee A, we have added a comprehensive discussion on the general applicability of the proposed methodology to other MGRS platforms in the revised Discussion section.

**Comment #18:**

[Line 152] should be past tense

**Reply:**

We agree with the referee.

**Changes:**

In response to Comment #36 by referee A, the highlighted statement has been revised and moved to Section 4.5.

**Comment #19:**

[Line 170] You have sometimes present and sometimes past tense - make consistent

**Reply:**

We agree with the referee.

**Changes:**

In response to Comment #36 by referee A, the highlighted statement has been revised and moved to Section 4.5.

**Comment #20:**

[Line 177–178] Strictly define all of these symbols before using them. We shouldn't see Xi_Cs-137 for the first time in an equation

**Reply:**

We agree with the referee.

**Changes:**

In response to Comment #36 by referee A, the highlighted statement has been revised and moved to Sections 4.3 and 4.5.

**Comment #21:**

[Figure 2] Distinguish lines by style (dash, dotted, ... ) as well as by colour. Rethink the orange and green on solid grey - it's hard to see even on a colour screen for a colour-sighted person. You can take the time in your caption to explain with more words the information that you give in the legend - i.e. write out the legend in sentences in the caption to increase comprehensibility.

**Reply:**

We agree with the referee that improved visual discrimination between curves is important for readability. We revised the figure by adopting perceptually uniform color maps for the natural and anthropogenic sources, which provide improved and more robust separation between curves across the full range of values.

In addition, we removed the gray shaded background to further enhance contrast and overall clarity. All information previously encoded in the shading remains fully specified in the formal model description (see also Comment #18 by referee A). We have carefully tested the adopted color schemes using the color-blindness simulator and confirm that they remain distinguishable for Protanopia, Deuteranopia, and Tritanopia.

In addition, we carefully tested the use of additional line styles but found that, given the number of curves and the limited set of clearly distinguishable line types, this approach introduced unwanted visual correlations between the different classes of sources and thereby reduced overall interpretability.

Regarding the caption, we note that the Nature Communications style guide encourages concise figure captions (within a strict word limit). We therefore retain a concise caption while ensuring all necessary information is clearly conveyed.

**Changes:**

The figure has been updated by improving the color scheme for better contrast and by introducing clearer visual separation between curves. The gray shaded region was removed, while ensuring that all prior information remains fully specified and accessible through the formal model description (see also Comment #18 by Referee A).

**Comment #22:**

[Figure 2] 'Statistical uncertainties .. " not "Uncertainties"

**Reply:**

We agree with the referee and have corrected the terminology accordingly.

**Changes:**

The label in Figure 2 was updated from "Uncertainties" to "Statistical uncertainties".

**Comment #23:**

[Figure 3] Red-green colour-blind is very common - need dashed, dotted, ... lines

**Reply:**

We agree with the referee that line styles and color contrast are important for readability and accessibility. The figures have therefore been revised accordingly. We have carefully tested the adopted color scheme using the color-blindness simulator and confirm that it remains distinguishable for Protanopia, Deuteranopia, and Tritanopia. The chosen color palette is consistent with color schemes commonly adopted in *Nature* journal publications and was selected as a compromise that provides good discrimination across all three common forms of color-vision deficiency.

**Changes:**

We have updated the figures by introducing distinct line styles (solid, dashed, dotted) in addition to color coding to improve clarity. The color scheme was retained but verified for accessibility, and the line styles in Fig. 3 were modified accordingly.

**Comment #24:**

[Line 219–221] precision could also be independently evaluated by re-performing your analysis for each of the 1-s and 5-s samples you have available and looking at the variability among all of these solutions

**Reply:**

We agree that evaluating the variability across multiple reconstructions could in principle provide additional insight into the stability of the inversion. However, we decided against this approach for two main reasons.

First, applying this procedure consistently across the full dataset is not feasible in a uniform way, as the number of available 1 s and 5 s samples differs between cases. As a result, any comparison of variability would depend on the specific segmentation strategy and would introduce a sampling-dependent bias in the inferred precision estimates rather than a consistent uncertainty metric across the dataset.

Second, the proposed procedure would quantify not only counting-statistical variability, but also additional variability arising from fluctuations in the experimental conditions between individual acquisition intervals. As discussed in our response to Comment #5 by referee A, MGRS measurements are subject to temporal variations in factors such as background radiation fields, environmental conditions, platform attitude, and detector response, resulting in overdispersion relative to an ideal stationary Poisson process. Consequently, the spread observed across repeated 1 s or 5 s inversions would reflect a mixture of counting-statistical and experimental variability. In contrast, the objective of the present analysis is precisely to evaluate the uncertainty associated with realistic 1 s, 5 s, and full-acquisition measurement scenarios, including these effects. For a more detailed discussion, we refer the referee to our response to Comment #31 by referee A.

For these reasons, we consider the posterior distribution to provide the most consistent and physically meaningful quantification of uncertainty for our application.

**Changes:**

No changes required.

**Comment #25:**

[Line 253] source strength

**Reply:**

We agree with the referee. In response to Comment #28 by referee A, the Discussion section has been restructured, during which the highlighted statement was removed.

**Changes:**

The corresponding statement has been removed as part of the restructuring of the Discussion section.

**Comment #26:**

[Line 257] radionuclide concentration quantification or radionuclide emission-rate quantification

**Reply:**

We agree with the referee. In response to Comment #28 by referee A, the Discussion section has been restructured, during which the highlighted statement was removed.

**Changes:**

The corresponding statement has been removed as part of the restructuring of the Discussion section.

## R3 Additional Changes

Here, we summarize all minor changes made in the revised manuscript and Supplementary Materials that have not been addressed in the responses above:

1. To improve reproducibility and foster open data research, we have expanded the shared experimental dataset and now also include the complete set of adopted spectral templates in the Zenodo repository [5] under accession code `https://doi.org/10.5281/zenodo.18004440`.
2. We improved the consistency of the measurement live-time notation by replacing $t_{\text{live}}$ with $t_{\text{gr}}$ throughout the manuscript.
3. We removed the outline boxes in Fig. 2 to improve visual clarity and reduce clutter across the affected panels.
4. Compliance of the presented figures has again been cross-checked against the journal's figure guidelines. Accordingly, we have replaced square bracket notation for units in figure axes with parentheses throughout the manuscript.
5. All figures and tables in the Supplementary Materials were updated to reflect changes made in the main manuscript.

All modifications are fully traceable in the attached LaTeX`diff` marked-up manuscript.

## R4 Bibliography


[1] J. D. Allyson and D. C. Sanderson. Monte Carlo simulation of environmental airborne gamma-spectrometry. *J. Environ. Radioact.*, 38(3):259–282, Jan. 1998. ISSN 0265931X. doi: 10.1016/S0265-931X(97)00040-4.

[2] M. S. Bandstra, D. Hellfeld, J. R. Vavrek, B. J. Quiter, K. Meehan, P. J. Barton, J. W. Cates, A. Moran, V. Negut, R. Pavlovsky, and T. H. Y. Joshi. Improved Gamma-Ray Point Source Quantification in Three Dimensions by Modeling Attenuation in the Scene. *IEEE Trans. Nucl. Sci.*, 68(11):2637–2646, Apr. 2021. doi: 10.1109/TNS.2021.3113588.

[3] D. Breitenmoser, F. Cerutti, G. Butterweck, M. M. Kasprzak, and S. Mayer. Emulator-based Bayesian inference on non-proportional scintillation models by compton-edge probing. *Nat. Commun.*, 14:7790, Nov. 2023. ISSN 2041-1723. doi: 10.1038/s41467-023-42574-y.

[4] D. Breitenmoser, A. Stabilini, M. M. Kasprzak, and S. Mayer. Development and validation of a high-fidelity full-spectrum Monte Carlo model for the Swiss airborne gamma-ray spectrometry system. *NIM-A*, 1077:170512, Aug. 2025. ISSN 0168-9002. doi: 10.1016/j.nima.2025.170512.

[5] D. Breitenmoser, A. Stabilini, M. M. Kasprzak, and S. Mayer. Quantitative mobile gamma-ray spectrometry through Bayesian inference (Dataset), Dec. 2025.

[6] D. Breitenmoser, R. Lopez, S. D. Clarke, and S. A. Pozzi. Identifying neutron sources using recoil and time-of-flight spectroscopy. *Phys. Rev. Appl.*, 25(6):064013, June 2026. doi: 10.1103/v6j6-f4rx.

[7] D. Breitenmoser, A. Stabilini, M. M. Kasprzak, and S. Mayer. Full-spectrum modeling of mobile gamma-ray spectrometry systems in scattering media. *Phys. Rev. Appl.*, 25(1):014044, Jan. 2026. doi: 10.1103/yhz2-xmby.

[8] S. P. Brooks and A. Gelman. General Methods for Monitoring Convergence of Iterative Simulations. *J. Comput. Graph. Stat.*, 7(4):434–455, Dec. 1998. ISSN 10618600. doi: 10.2307/1390675.

[9] J. Buchner, T. Boller, D. Bogensberger, A. Malyali, K. Nandra, J. Wilms, T. Dwelly, and T. Liu. Systematic evaluation of variability detection methods for eROSITA. *A&A*, 661:A18, May 2022. ISSN 0004-6361, 1432-0746. doi: 10.1051/0004-6361/202141099.

[10] G. Butterweck, B. Bucher, D. Breitenmoser, L. Rybach, C. Poretti, S. Maillard, M. Kasprzak, G. Ferreri, A. Gurtner, M. Astner, F. Hauenstein, M. Straub, M. Bucher, C. Harm, G. Scharding, and S. Mayer. Aeroradiometric measurements in the framework of the Swiss exercise ARM20. Technical report, Paul Scherrer Institut (PSI), Villigen PSI, Switzerland, 2021.

[11] J. C. Curtis, R. J. Cooper, T. H. Joshi, B. Cosofret, T. Schmit, J. Wright, J. Rameau, D. Konno, D. Brown, F. Otsuka, E. Rappeport, M. Marshall, and J. Speicher. Simulation and validation of the Mobile Urban Radiation Search (MURS) gamma-ray detector response. *Nucl. Instrum. Methods Phys. Res. Sect. Accel. Spectrometers Detect. Assoc. Equip.*, 954:161128, Feb. 2020. ISSN 01689002. doi: 10.1016/j.nima.2018.08.087.

[12] J. N. Fry and S. Colombi. Void statistics and hierarchical scaling in the halo model. *MNRAS*, 433(1):581–590, July 2013. ISSN 0035-8711. doi: 10.1093/mnras/stt745.

[13] A. Gelman, J. B. Carlin, H. S. Stern, D. B. Dunson, A. Vehtari, and D. B. Rubin. *Bayesian Data Analysis*. Chapman and Hall/CRC, New York, USA, 3rd edition, Nov. 2013. ISBN 978-0-429-11307-9. doi: 10.1201/B16018.

[14] R. L. Grasty, P. B. Holman, and Y. B. Blanchard. Transportable calibration pads for ground and airborne gamma-ray spectrometers. *Geol. Surv. Can.*, 90(23), 1991. doi: 10.4095/132237.

[15] M. Hameeda, A. Plastino, and M. C. Rocca. Generalized Poisson distributions for systems with two-particle interactions. *IOPSciNotes*, 2(1):015003, Mar. 2021. ISSN 2633-1357. doi: 10.1088/2633-1357/abec9f.

[16] D. Hellfeld, M. S. Bandstra, J. R. Vavrek, D. L. Gunter, J. C. Curtis, M. Salathe, R. Pavlovsky, V. Negut, P. J. Barton, J. W. Cates, B. J. Quiter, R. J. Cooper, K. Vetter, and T. H. Joshi. Free-moving Quantitative Gamma-ray Imaging. *Sci. Rep.*, 11:20515, Oct. 2021. ISSN 2045-2322. doi: 10.1038/s41598-021-99588-z.

[17] M. Hünnefeld, R. Abbasi, M. Ackermann, J. Adams, J. A. Aguilar, M. Ahlers, M. Ahrens, C. Alispach, A. A. Alves, N. M. Amin, R. An, K. Andeen, T. Anderson, G. Anton, C. Argüelles, Y. Ashida, S. Axani, X. Bai, A. V. Balagopal, A. Barbano, S. W. Barwick, B. Bastian, V. Basu, S. Baur, R. Bay, J. J. Beatty, K. H. Becker, J. Becker Tjus, C. Bellenghi, S. BenZvi, D. Berley, E. Bernardini, D. Z. Besson, G. Binder, D. Bindig, E. Blaufuss, S. Blot, M. Boddenberg, F. Bontempo, J. Borowka, S. Böser, O. Botner, J. Böttcher, E. Bourbeau, F. Bradascio, J. Braun, S. Bron, J. Brostean-Kaiser, S. Browne, A. Burgman, R. T. Burley, R. S. Busse, M. A. Campana, E. G. Carnie-Bronca, C. Chen, D. Chirkin, K. Choi, B. A. Clark, K. Clark, L. Classen, A. Coleman, G. H. Collin, J. M. Conrad, P. Coppin, P. Correa, D. F. Cowen, R. Cross, C. Dappen, P. Dave, C. De Clercq, J. J. DeLaunay, H. Dembinski, K. Deoskar, S. De Ridder, A. Desai, P. Desiati, K. D. de Vries, G. de Wasseige, M. de With, T. DeYoung, S. Dharani, A. Diaz, J. C. Díaz-Vélez, M. Dittmer, H. Dujmovic, M. Dunkman, M. A. DuVernois, E. Dvorak, T. Ehrhardt, P. Eller, R. Engel, H. Erpenbeck, J. Evans, P. A. Evenson, K. L. Fan, A. R. Fazely, S. Fiedlschuster, A. T. Fienberg, K. Filimonov, C. Finley, L. Fischer, D. Fox, A. Franckowiak, E. Friedman, A. Fritz, P. Fürst, T. K. Gaisser, J. Gallagher, E. Ganster, A. Garcia, S. Garrappa, L. Gerhardt, A. Ghadimi, C. Glaser, T. Glauch, T. Glüsenkamp, A. Goldschmidt, J. G. Gonzalez, S. Goswami, D. Grant, T. Grégoire, S. Griswold, M. Gündüz, C. Günther, C. Haack, A. Hallgren, R. Halliday, L. Halve, F. Halzen, M. Ha Minh, K. Hanson, J. Hardin, A. A. Harnisch, A. Haungs, S. Hauser, D. Hebecker, K. Helbing, F. Henningsen, E. C. Hettinger, S. Hickford, J. Hignight, C. Hill, G. C. Hill, K. D. Hoffman, R. Hoffmann, T. Hoinka, B. Hokanson-Fasig, K. Hoshina, F. Huang, M. Huber, T. Huber, K. Hultqvist, R. Hussain, S. In, N. Iovine, A. Ishihara, M. Jansson, G. S. Japaridze, M. Jeong, B. J. Jones, D. Kang, W. Kang, X. Kang, A. Kappes, D. Kappesser, T. Karg, M. Karl, A. Karle, U. Katz, M. Kauer, M. Kellermann, J. L. Kelley, A. Kheirandish, K. Kin, T. Kintscher, J. Kiryluk, S. R. Klein, R. Koirala, H. Kolanoski, T. Kontrimas, L. Köpke, C. Kopper, S. Kopper, D. J. Koskinen, P. Koundal, M. Kovacevich, M. Kowalski, T. Kozynets, E. Kun, N. Kurahashi, N. Lad, C. Lagunas Gualda, J. L. Lanfranchi, M. J. Larson, F. Lauber, J. P. Lazar, J. W. Lee, K. Leonard, A. Leszczyńska, Y. Li, M. Lincetto, Q. R. Liu, M. Liubarska, E. Lohfink, C. J. Lozano Mariscal, L. Lu, F. Lucarelli, A. Ludwig, W. Luszczak, Y. Lyu, W. Y. Ma, J. Madsen, K. B. Mahn, Y. Makino, S. Mancina, I. C. Mariş, R. Maruyama, K. Mase, T. McElroy, F. McNally, J. V. Mead, K. Meagher, A. Medina, M. Meier, S. Meighen-Berger, J. Micallef, D. Mockler, T. Montaruli, R. W. Moore, R. Morse, M. Moulai, R. Naab, R. Nagai, U. Naumann, J. Necker, L. V. Nguyên, H. Niederhausen, M. U. Nisa, S. C. Nowicki, D. R. Nygren, A. Obertacke Pollmann, M. Oehler, A. Olivas, E. O'Sullivan,

H. Pandya, D. V. Pankova, N. Park, G. K. Parker, E. N. Paudel, L. Paul, C. Pérez de los Heros, L. Peters, J. Peterson, S. Philippen, D. Pieloth, S. Pieper, M. Pittermann, A. Pizzuto, M. Plum, Y. Popovych, A. Porcelli, M. Prado Rodriguez, P. B. Price, B. Pries, G. T. Przybylski, C. Raab, A. Raissi, M. Rameez, K. Rawlins, I. C. Rea, A. Rehman, P. Reichherzer, R. Reimann, G. Renzi, E. Resconi, S. Reusch, W. Rhode, M. Richman, B. Riedel, E. J. Roberts, S. Robertson, G. Roellinghoff, M. Rongen, C. Rott, T. Ruhe, D. Ryckbosch, D. Rysewyk Cantu, I. Safa, J. Saffer, S. E. Sanchez Herrera, A. Sandrock, J. Sandroos, M. Santander, S. Sarkar, S. Sarkar, K. Satalecka, M. Scharf, M. Schaufel, H. Schieler, S. Schindler, P. Schlunder, T. Schmidt, A. Schneider, J. Schneider, F. G. Schröder, L. Schumacher, G. Schwefer, S. Sclafani, D. Seckel, S. Seunarine, A. Sharma, S. Shefali, M. Silva, B. Skrzypek, B. Smithers, R. Snihur, J. Soedingrekso, D. Soldin, C. Spannfellner, G. M. Spiczak, C. Spiering, J. Stachurska, M. Stamatikos, T. Stanev, R. Stein, J. Stettner, A. Steuer, T. Stezelberger, T. Stürwald, T. Stuttard, G. W. Sullivan, I. Taboada, F. Tenholt, S. Ter-Antonyan, S. Tilav, F. Tischbein, K. Tollefson, L. Tomankova, C. Tönnis, S. Toscano, D. Tosi, A. Trettin, M. Tselengidou, C. F. Tung, A. Turcati, R. Turcotte, C. F. Turley, J. P. Twagirayezu, B. Ty, M. A. Unland Elorrieta, N. Valtonen-Mattila, J. Vandenbroucke, N. van Eijndhoven, D. Vannerom, J. van Santen, S. Verpoest, M. Vraeghe, C. Walck, T. B. Watson, C. Weaver, P. Weigel, A. Weindl, M. J. Weiss, J. Weldert, C. Wendt, J. Werthebach, M. Weyrauch, N. Whitehorn, C. H. Wiebusch, D. R. Williams, M. Wolf, K. Woschnagg, G. Wrede, J. Wulff, X. W. Xu, Y. Xu, J. P. Yanez, S. Yoshida, S. Yu, T. Yuan, and Z. Zhang. Combining Maximum-Likelihood with Deep Learning for Event Reconstruction in IceCube. *Proc. Sci.*, 395:1065, Mar. 2022. ISSN 18248039. doi: 10.22323/1.395.1065.

[18] L. Hurtado-Gil, V. J. Martínez, P. Arnalte-Mur, M.-J. Pons-Bordería, C. Pareja-Flores, and S. Paredes. The best fit for the observed galaxy counts-in-cell distribution function. *A&A*, 601:A40, May 2017. ISSN 0004-6361, 1432-0746. doi: 10.1051/0004-6361/201629097.

[19] S. Kobayashi, N. Hasebe, E. Shibamura, O. Okudaira, M. Kobayashi, N. Yamashita, Y. Karouji, M. Hareyama, K. Hayatsu, C. D'Uston, S. Maurice, O. Gasnault, O. Forni, B. Diez, R. C. Reedy, and K. J. Kim. Determining the absolute abundances of natural radioactive elements on the lunar surface by the kaguya gamma-ray spectrometer. *Space Sci. Rev.*, 154(1-4):193–218, July 2010. ISSN 00386308. doi: 10.1007/s11214-010-9650-2.

[20] J. A. Kulisek, R. S. Wittman, E. A. Miller, W. J. Kernan, J. D. McCall, R. J. McConn, J. E. Schweppe, C. E. Seifert, S. C. Stave, and T. N. Stewart. A 3D simulation look-up library for real-time airborne gamma-ray spectroscopy. *Nucl. Instrum. Methods Phys. Res. Sect. Accel. Spectrometers Detect. Assoc. Equip.*, 879:84–91, Jan. 2018. ISSN 01689002. doi: 10.1016/j.nima.2017.10.030.

[21] C. Lyons and D. Colton. Aerial Measuring System in Japan. *Health Phys.*, 102(5):509, May 2012. ISSN 0017-9078. doi: 10.1097/HP.0b013e31824d0056.

[22] B. R. S. Minty, M. P. Morse, and L. M. Richardson. Portable Calibration Sources For Airborne Gamma-ray Spectrometers. *Explor. Geophys.*, 21(4):187–195, 1990. doi: 10.1071/EG990187.

[23] P. N. Peplowski. The global elemental composition of 433 Eros: First results from the NEAR gamma-ray spectrometer orbital dataset. *Planet. Space Sci.*, 134:36–51, Dec. 2016. ISSN 00320633. doi: 10.1016/j.pss.2016.10.006.

[24] L. A. Perez, S. Malhotra, J. E. Rhoads, and V. Tilvi. Void Probability Function of Simulated Surveys of High-redshift Ly$\alpha$ Emitters. *ApJ*, 906(1):58, Jan. 2021. ISSN 0004-637X. doi: 10.3847/1538-4357/abc88b.

[25] M. Praszalowicz. Negative Binomial Distribution and the multiplicity moments at the LHC. *Phys. Lett. B*, 704(5):566–569, Oct. 2011. ISSN 0370-2693. doi: 10.1016/j.physletb.2011.09.101.

[26] M. Salathe, B. J. Quiter, M. S. Bandstra, J. C. Curtis, R. Meyer, and C. H. Chow. Determining urban material activities with a vehicle-based multi-sensor system. *Phys. Rev. Res.*, 3(2): 023070, Apr. 2021. ISSN 26431564. doi: 10.1103/PHYSREVRESEARCH.3.023070.

[27] Y. Sanada, T. Sugita, Y. Nishizawa, A. Kondo, and T. Torii. The aerial radiation monitoring in Japan after the Fukushima Daiichi nuclear power plant accident. *Nucl. Sci. Technol.*, 4:76–80, 2014. doi: 10.15669/pnst.4.76.

[28] M. F. Santarelli, V. Positano, and L. Landini. Measured PET Data Characterization with the Negative Binomial Distribution Model. *J. Med. Biol. Eng.*, 37(3):299–312, June 2017. ISSN 2199-4757. doi: 10.1007/s40846-017-0236-2.

[29] S. V. Tezlaf. Significance of the negative binomial distribution in multiplicity phenomena. *Phys. Scr.*, 98(11):115310, Oct. 2023. ISSN 1402-4896. doi: 10.1088/1402-4896/acfead.

[30] J.-A. Tröger, U. Römer, and S. Hartmann. Comparing frequentist and Bayesian uncertainty quantification in two-step constitutive model calibration. *Proc. Appl. Math. Mech.*, 24(2): e202400031, 2024. ISSN 1617-7061. doi: 10.1002/pamm.202400031.

[31] R. Trotta. Bayes in the sky: Bayesian inference and model selection in cosmology. *Contemp. Phys.*, 49(2):71–104, Mar. 2008. ISSN 00107514. doi: 10.1080/00107510802066753.

[32] A. N. Vasil'Ev and A. V. Gektin. Multiscale approach to estimation of scintillation characteristics. *IEEE Trans. Nucl. Sci.*, 61(1):235–245, 2014. ISSN 00189499. doi: 10.1109/TNS.2013.2282117.

[33] U. von Toussaint. Bayesian inference in physics. *Rev. Mod. Phys.*, 83(3):943–999, Sept. 2011. ISSN 0034-6861. doi: 10.1103/RevModPhys.83.943.

[34] I. Winkelmann, C. Strobl, and M. Thomas. Aerial measurements of artificial radionuclides in Germany in case of a nuclear accident. *J. Environ. Radioact.*, 72(1-2):225–231, Jan. 2004. ISSN 0265931X. doi: 10.1016/S0265-931X(03)00205-4.

# Peer Review Report for

## Quantitative mobile gamma-ray spectrometry through Bayesian inference

David Breitenmoser[1,2,*], Alberto Stabilini[1], Malgorzata Magdalena Kasprzak[1], Sabine Mayer[1]

[1]Department of Radiation Safety and Security, Paul Scherrer Institute (PSI), Forschungsstrasse 111, Villigen PSI, 5232, Switzerland

[2]Department of Nuclear Engineering & Radiological Sciences, University of Michigan, 2355 Bonisteel Blvd., Ann Arbor, MI 48109-2104, United States of America

[*]Lead and contact author: David Breitenmoser (E-Mail: david.breitenmoser@psi.ch, ORCID: 0000-0003-0339-6592)

## Preface

We would like to thank the referees and editor for their important work and are grateful for their comments and suggestions. We believe that the proposed revisions have improved the manuscript and made it more complete. In this document, we carefully address all comments and describe the corresponding changes in the revised manuscript. For the readers' convenience, the referee's comments and our statements are color-coded (black: referee comment, blue: author reply). Line references in referee's comments are all with respect to the original manuscript. A marked-up manuscript highlighting all changes using LaTeX`diff` is attached to this revised submission.

# Contents

R1 Response to Referee A . . . . . R1
R2 Response to Referee B . . . . . R12
R3 Additional Changes . . . . . R13
R4 Bibliography . . . . . R15

## R1 Response to Referee A

**Comment #1:**

I have a few major but subtle concerns left that I believe can be relatively easily addressed. The first concerns how the authors frame the motivation for their method. Throughout the paper (particularly in the Introduction and Discussion), the authors repeatedly argue for their Bayesian approach by claiming what classical Poisson or Gaussian MLE methods cannot do (stating that they produce biased estimates, yield unstable solutions, or fail with sparse statistics). However, because the authors do not actually run a direct side-by-side comparative benchmark against a standard frequentist/MLE pipeline on this dataset, these claims about the limitations of Poisson/MLE feel unsubstantiated. I agree that the presented Bayesian method is very powerful and well founded on its own terms. I suggest the authors reframe these statements: instead of claiming classical Poisson/MLE fails at X, simply point out what the Bayesian framework naturally provides (such as principled uncertainty quantification, direct access to parameter correlations, flexible handling of overdispersed likelihoods, and robust treatment of nuisance parameters).

**Reply:**

We thank the referee for this helpful suggestion. We agree that the original wording could be interpreted as making direct performance claims regarding frequentist maximum-likelihood approaches without providing a dedicated benchmark in the present work. Following the referee's recommendation, we have revised the relevant passages in the manuscript to avoid such comparisons and instead emphasize the inherent capabilities and advantages of the proposed Bayesian framework, including principled uncertainty quantification, joint inference of correlated parameters, flexible treatment of nuisance parameters, and the ability to accommodate more general likelihood models when appropriate.

**Changes:**

The relevant passages have been revised throughout to remove unsupported comparative statements regarding frequentist/MLE approaches and to instead focus on the methodological capabilities and motivation for the proposed Bayesian framework. Statements supported by prior work and established findings have been retained where appropriate. All modifications are fully traceable in the attached LaTeX`diff` marked-up manuscript.

**Comment #2:**

The second is regarding $\alpha_{\mathrm{NB}}$ and how the authors argue its behavior. I believe what they observe has a simple explanation. Relative statistical uncertainties scale as $1/\sqrt(N)$, so at low statistics any modeling artifact (overdispersion, pileup, template inaccuracy) is simply buried in the noise. Once the statistics increase, the relative uncertainty drops quickly and tension with the model becomes measurable. I have seen this in my own work many times: at low statistics a model describes the data very well, but when the integration time is increased the fit degrades. $\alpha_{\mathrm{NB}}$ can absorb some of this tension, and at the 5 min integration time it is required to do so. I do not believe that small variations over 5 minutes lead to additional overdispersion, as implied at line 215, but rather that at 5 min there are sufficient statistics to sense any model deficiency. While the authors mostly follow this line of thought, I believe they can improve the language around it. In particular, the statement at lines 217–220 seems to argue the opposite. Modeling gamma-ray transport in unconstrained environments is excruciatingly hard, and this work does it about as well as I have seen. Clearly stating the limits of one's approach does not weaken a publication but strengthens it.

**Reply:**

We agree with the referee that the original wording could be interpreted as attributing the increase in $\alpha_{\mathrm{NB}}$ directly to time-dependent overdispersion effects, which is not uniquely supported by the data. The referee's interpretation provides a more appropriate perspective: as statistical uncertainties decrease with increasing acquisition time, previously hidden discrepancies between the model and observations may become increasingly detectable. We have therefore revised the discussion to clarify that the inferred dispersion parameter captures departures from the ideal Poisson model, which may originate from residual model inaccuracies, unmodeled measurement effects, or other sources of variability, rather than necessarily indicating an intrinsic increase in overdispersion with integration time.

**Changes:**

The highlighted text passage has been revised as follows:

> "This behavior indicates increasing sensitivity to departures from the ideal Poisson counting model as the statistical uncertainty decreases with longer acquisition times. At short integration times, statistical fluctuations may dominate over small model discrepancies, whereas longer acquisitions provide sufficient counting statistics to reveal residual inconsistencies between the observations and the assumed forward model. While the forward model accounts for dominant second-order environmental effects, including atmospheric contributions and ground back-scattering (see Section 4.3), residual effects such as higher-order scattering contributions, detector response deviations, or other unmodeled measurement variations may contribute to the observed trend."

**Comment #3:**

The last is about modeling uncertainties. The authors largely assume the model inputs are exact, while acknowledging elsewhere that the GNSS positioning, for example, carries non-negligible uncertainty. I recognize that handling these properly is well beyond the scope of the current work: they are not amenable to simple downstream error propagation, since they enter at the template-generation stage. It would be good, however, to state this limitation explicitly and adding full propagation of D's uncertainties to the Discussion as a potential direction for future work would also be reasonable.

**Reply:**

We agree with the referee that uncertainties in the experimental conditions $\mathfrak{D}$, such as platform position and orientation, are not propagated in the present work and that their treatment requires extending the forward-model generation rather than applying simple downstream error propagation. We have therefore added an explicit discussion of this limitation and identified the propagation of uncertainties in $\mathfrak{D}$ through the forward model and Bayesian inference framework as an important direction for future work.

**Changes:**

We added the following paragraph in the Discussion section:

> "A second important extension concerns the treatment of uncertainty in the known experimental conditions $\mathfrak{D}$. In the present work, platform position, orientation, and other

> measurement-state parameters are treated as deterministic inputs during template generation. In practice, these quantities are themselves subject to measurement uncertainties. Future developments could explicitly propagate these uncertainties through the forward-model generation and Bayesian inference framework, enabling a more complete characterization of model uncertainty and its impact on the inferred source parameters under realistic operational conditions."

**Comment #4:**

Section 2.1 mentions that a template evaluation takes O(1) s, and line 114 gives O($10^4$) likelihood evaluations, but the resulting end-to-end runtime is never stated. It is important to provide context on whether an inference runs in minutes, hours, or days; please add a typical wall-clock runtime. The method is presented as much faster than traditional Monte Carlo approaches, but relies on $5.5 \times 10^4$ core-hours for the IRF set and $\sim 10^2$ core-hours per source for the flux libraries. The upfront cost is therefore high, comparable to what is quoted for a single brute-force evaluation, but the results are reusable, and that is worth mentioning as well.

**Reply:**

We thank the referee for this helpful suggestion. We have clarified the distinction between the one-time computational cost of generating the high-fidelity forward-model components and the computational cost of the Bayesian inversion itself. Specifically, we now state that the precomputed IRFs can be reused across multiple inference tasks and report the typical wall-clock runtime of a complete Bayesian inversion.

**Changes:**

In Section 4.2, we added:

> "These precomputed IRFs represent a one-time computational investment for a given detector and platform configuration and can subsequently be reused across multiple template-generation and Bayesian inference tasks, thereby avoiding repeated high-fidelity radiation-transport simulations while preserving the accuracy of the underlying forward model."

In Section 4.6, we added:

> "The computational cost of the Bayesian inversion remained moderate, with a complete MCMC run requiring $\mathcal{O}(10^2)$ s wall-clock time on a single Intel Xeon Gold 6130 processor core ($2.1\,\mathrm{GHz}$)."

**Comment #5:**

Section 4.3 needs to better discuss the flux scoring methodology. Furthermore, Ref. [40] appears not to include a ground plane, so it is necessary to be precise here about how the distance of the detector from the ground plane (30 m and 90 m) is accounted for, and whether it is generally assumed that the helicopter does not change altitude during the encounter.

**Reply:**

We agree with the referee that the flux scoring methodology and treatment of ground clearance in Section 4.3 were not described with sufficient detail to ensure reproducibility. We have revised the manuscript to clarify that the ground clearance is accounted for dynamically for each spectrum instance in both hover-flight and single-pass measurements, without assuming a constant helicopter altitude. The corresponding flux libraries were interpolated from the precomputed ground-clearance grid during spectral template generation.

**Changes:**

In Section 4.3, we added the following statement:

> "The double-differential flux was evaluated on a grid of ground clearances from $10\,\mathrm{m}$ to $250\,\mathrm{m}$ in $10\,\mathrm{m}$ increments using the reciprocal transform method [1–3]. The resulting flux libraries were subsequently interpolated to the recorded platform ground clearance for each spectrum instance during spectral template generation (see also Section 4.5)."

In Section 4.2, we added the following statement:

> "In the present work, $\mathfrak{d}$ comprises the dynamic helicopter pose relative to the ground, fuel state, and ambient environmental conditions (air temperature, pressure, and humidity), as introduced in Section 2.1."

Furthermore, Section 4.5 describes how these experimental conditions are updated for each spectrum instance:

> "The experimental conditions $\mathfrak{D}$ are evaluated (or interpolated where required) at the corresponding spectrum instances $k = 1, \ldots, N_{\mathcal{Y}}$ via the mapping $\mathfrak{d}_k \leftrightarrow t_k$, thereby linking each measured spectrum to its associated acquisition conditions."

**Comment #6:**

Section 4.4 describes four independently read-out crystals, but Section 2.2 mentions a single volume. Please simply state in Section 2.2 that the four independent crystals are effectively used as a single, spatially unresolved detector.

**Reply:**

We agree and have clarified in Section 2.2 that the four independently read-out crystals are combined into a single detector spectrum for the present analysis, with an additional cross-reference pointing to the related Methods section.

**Changes:**

In the data-reduction paragraph of Section 2.2, we added:

> "The four independently read-out scintillation crystals were treated jointly as a single detector response in the present analysis, [...] (see Section 4.4)."

**Comment #7:**

The abstract could be improved slightly. First, I would carefully weigh the use of "sources" when referring to radioactive material in general.

**Reply:**

We thank the referee for this helpful suggestion. We have revised the terminology in the abstract, replacing "sources" with "emitters" where appropriate and distinguishing more clearly between radioactive material and the more general class of gamma-ray emitters. We have also avoided combining "radionuclide" and "source" into a single term (e.g., "radionuclide sources"), instead referring separately to radionuclides, radioactive materials, or gamma-ray sources, as appropriate to the specific context.

**Changes:**

The terminology in the abstract was revised accordingly. As LaTeXdiff does not allow changes in the Abstract to be highlighted, we reproduce the revised Abstract in full below:

> "Accurate quantitative mapping of gamma-ray emitters is critical for applications ranging from radiological emergency response and environmental monitoring to nuclear security and deep space exploration. Here, we show that such mapping can be achieved by combining mobile gamma-ray spectrometry with high-fidelity Monte Carlo simulations and full-spectrum Bayesian inference. Using 6 s of single-pass mobile spectrometry data benchmarked against independent *in-situ* and laboratory assays, we demonstrate decisive source mixture identification ($>5\sigma$), meter-scale source localization, and recovery of source activities with percent-level accuracy. The developed method marks a critical advance in quantitative gamma-ray sensing, enabling improved radiological situational awareness, enhanced terrestrial geophysical and geochemical mapping, as well as more robust constraints on radionuclide abundances on extraterrestrial bodies across the Solar System."

**Comment #8:**

Second, I personally do not understand what "platform-dynamic" means.

**Reply:**

The term "platform-dynamic" was intended to indicate that the Monte Carlo mass model accounts for changes in platform-state parameters, e.g. the fuel level. We agree that the term is ambiguous without further context and have therefore removed it from the abstract.

**Changes:**

The term "platform-dynamic" was removed from the abstract.

**Comment #9:**

Third, you do not present source localization of distributed sources; one could argue that the backgrounds are distributed, but the phrasing implies something different.

**Reply:**

We agree with the referee that the original wording could be interpreted as implying source localization for distributed sources. We have therefore generalized the first sentence to describe the framework in terms of the quantitative inference, without explicitly distinguishing between distributed and point-like sources at this stage. We then specify the demonstrated capabilities and their respective performance in the following sentence.

**Changes:**

In the Abstract, we revised the corresponding part to:

> "Here, we show that such mapping can be achieved by combining mobile gamma-ray spectrometry with high-fidelity Monte Carlo simulations and full-spectrum Bayesian inference. Using 6 s of single-pass mobile spectrometry data benchmarked against independent *in-situ* and laboratory assays, we demonstrate decisive source mixture identification ($>5\sigma$), meter-scale source localization, and recovery of source activities with percent-level accuracy."

**Comment #10:**

Fourth, the claim of  1% error in 1 s is a bit strong given the results, which are more on the order of 2–4%.

**Reply:**

We agree that the previous wording overstated the accuracy demonstrated by the validation results. We have therefore replaced the "$\sim$ 1% error" statement with the more conservative "percent-level accuracy."

**Changes:**

In the Abstract, "$\sim$ 1% error" was replaced with "percent-level accuracy".

**Comment #11:**

Finally, the statement that this method quantifies anthropogenic sources that conventional methods cannot resolve would require proof that they are indeed inaccessible to classical methods. The sources here are relatively strong and clearly visible in the spectra, so I do not think even the claim of particular sensitivity is substantiated. At minimum, please add a comma after "resolve", as the phrase is currently ambiguous regarding what the 1 s and  1% error refer to.

**Reply:**

We agree that the original statement was too broad. We have removed the comparative claim.

**Changes:**

We rephrased the highlighted statement in the abstract as follows:

> "Using 6 s of single-pass mobile spectrometry data benchmarked against independent *in-situ* and laboratory assays, we demonstrate decisive source mixture identification ($>5\sigma$), meter-scale source localization, and recovery of source activities with percent-level accuracy."

**Comment #12:**

Section 2.1: It would help to explain the experimental conditions here rather than at lines 167–168, as the concept is otherwise a bit too abstract. The same is true for the source mixture S; you could explain here that this is simply an assumption about which and how many sources are present.

**Reply:**

Regarding the experimental conditions, we agree that the original definition was somewhat abstract at this point in the manuscript. We intentionally keep $\mathfrak{d}$ general so that the formalism is not restricted to the specific state parameters considered in this study. As a compromise, we have added representative examples to clarify the meaning of this parameter without limiting its generality.

For the source mixture, we have clarified the definition to make explicit that $\mathcal{S}$ specifies the assumed set of sources, including their number and identities.

**Changes:**

For the experimental conditions, we revised the corresponding sentence to:

> "The corresponding acquisition is carried out under known experimental conditions $\mathfrak{d}_t \in \mathbb{R}^{N_\mathfrak{d}}$, which encode the known platform and environmental state parameters, e.g., the platform position and atmospheric pressure, with the full set of $N_\mathfrak{D} \in \mathbb{N}$ experimental conditions denoted as $\mathfrak{D} = \{\mathfrak{d}_t \in \mathbb{R}^{N_\mathfrak{d}}\}_{t=1}^{N_\mathfrak{D}}$."

For the source mixture, we revised the definition to clarify that it represents the assumed set of sources as follows:

> "Within this Bayesian FSA formalism, the inverse problem consists of jointly inferring the gamma-ray source strengths $\Xi = \{\xi_s \in \mathbb{R}_+\}_{s=1}^{N_s}$ and source locations $\mathcal{X} = \{\mathbf{x}_s \in \mathbb{R}^3\}_{s=1}^{N_s}$ for an assumed source mixture $\mathcal{S}$, comprising $N_s \in \mathbb{N}$ sources whose identities are specified a priori."

**Comment #13:**

Section 2.3 should state right in the first paragraph that the source location is not estimated here. It is currently only implied in the following paragraph.

**Reply:**

We agree and have clarified this explicitly in the opening paragraph of Section 2.3.

**Changes:**

We added:

> "We first evaluate the proposed Bayesian inversion framework under the controlled hover-flight configuration, restricting the inference to radionuclide activities while fixing the source locations to their known experimental positions."

**Comment #14:**

Overall, I would describe the dimensionality of the problem better. How many bins per spectrum are used, and how many spectra (1 or 6, I believe)? The data are quite limited, which makes the accuracy the more impressive.

**Reply:**

We agree that the dimensionality of the measurement data should be stated more explicitly. We have therefore added the number of spectra and spectral channels used in the two benchmark configurations.

**Changes:**

In Section 2.2, we now state:

> "Each dataset comprises a set of gross-count spectra $\mathcal{Y}$ ($N_{\mathcal{Y}} = 1$ for the hover-flight benchmark and $N_{\mathcal{Y}} = 6$ for the single-pass benchmark) with associated measurement live times $\Delta t_{\mathrm{gr}}$ and a set of known experimental conditions $\mathfrak{D}$ that encodes the platform and environmental states relevant to the likelihood evaluation: helicopter position and orientation (pose), fuel level, and ambient conditions (air temperature, pressure, humidity)."

We further clarified the number of spectral channels evaluated in Section 2.2:

> "The four independently read-out scintillation crystals were treated jointly as a single detector response in the present analysis, yielding spectra with 1008 channels spanning an energy range from $\sim$50 keV to $\sim$3 MeV (see Section 4.4)."

For consistency, we also revised the related system description in Section 4.4 as follows:

> "Each spectrum comprises 1008 channels spanning $\sim$50 keV to $\sim$3 MeV."

**Comment #15:**

Panel c in Figs. 4–6 is extremely complex. A bit more context on how to interpret the various numbers would be valuable, and it may help to mention that the model index is always ordered from least likely to most likely.

**Reply:**

We agree that additional context would improve the interpretation of panel c. We have therefore clarified the meaning of the reported quantities, explicitly stated the ordering of the source-mixture models, as well as provided a cross-reference to the section containing the formal definition of the displayed quantities.

**Changes:**

In the captions of Figs. 4–6, we added:

> "Bayesian model comparison results. The anti-diagonal entries show the log Bayesian evidence $\log \mathcal{Z}_{i=j}$ for each source-mixture model (see Section 2.1). The models are ordered by increasing Bayesian evidence, from the least supported at the bottom left to the most supported at the top right. The entries above the anti-diagonal show the pairwise log Bayes factors $\log \mathcal{B}_{ij} = \log \mathcal{Z}_i - \log \mathcal{Z}_j$, while those below the anti-diagonal show the corresponding lower bounds on statistical significance, expressed as Gaussian-equivalent significance levels in units of standard deviations [4, 5]. Related uncertainties are reported as one standard errors using least-significant-figure notation."

**Comment #16:**

O(X) notation: I do not see why it is needed in most places, but if you wish to keep it, please use O(10) m, O(100) m, O(100) km, which conveys the scale directly. O($10^5$) m takes me 30 seconds to work out.

**Reply:**

We have adopted the suggested notation throughout the manuscript.

**Changes:**

The notation has been revised throughout the manuscript accordingly. All modifications are fully traceable in the attached LaTeX`diff` marked-up manuscript.

**Comment #17:**

Line 201: I think the best estimates and uncertainties should be added to Fig. 3 or mentioned here explicitly. Otherwise, the statement is not verifiable.

**Reply:**

We thank the referee for this suggestion. For the inferred source activities, we agree that the individual posterior estimates and their uncertainties are not readily compared from Fig. 3 alone. We intentionally did not list all six individual estimates in the main text, as this would make the presentation unnecessarily cumbersome. Instead, we have provided the full posterior activity estimates and uncertainties for each measurement in Supplementary Tables S3 and S4 and added corresponding cross-references, making the quantitative comparison underlying the statement explicitly verifiable.

**Changes:**

We added a cross-reference to the supplementary materials:

> "Nevertheless, for all experiments, the posterior activity estimates are statistically consistent with the corresponding *in-situ* constraints as well as with each other within their combined uncertainty ranges (see Supplementary Tables S3 and S4)."

**Comment #18:**

There are two or three different notations for the natural radon component; please choose one.

**Reply:**

We thank the referee for this comment. We agree that the distinction between the two notations ($\Delta\mathrm{Rn}_{\mathrm{nat}}$ and $\Delta\xi_{\text{Rn-nat}}$) is not necessary for the present analysis and could lead to unnecessary confusion. We have therefore removed the notation $\Delta\mathrm{Rn}_{\mathrm{nat}}$ entirely and consistently retained only the notation for the associated radon activity concentration, $\Delta\xi_{\text{Rn-nat}}$.

**Changes:**

The notation $\Delta\mathrm{Rn}_{\mathrm{nat}}$ was removed throughout the manuscript and Supplementary Materials. All modifications are fully traceable in the attached LaTeX`diff` marked-up manuscript.

**Comment #19:**

The posterior in Fig. 2 is a bit hard to see, and given that the templates are histograms, step functions could be used.

**Reply:**

We agree with the referee that the posterior shading was difficult to distinguish. We have therefore increased its opacity. We also evaluated a step-function representation of the templates. However, given the $\sim 10^3$ spectral channels, the closely spaced steps visually merge at the scale of the figure and reduce rather than improve readability. We therefore retained the continuous representation of the templates, also consistent with the spectral density normalization adopted in the highlighted figure.

**Changes:**

The opacity of the posterior shading in Fig. 2 was increased.

**Comment #20:**

Finally, I would like to applaud the authors for carrying out such a challenging analysis with this level of rigor, and for producing a truly excellent publication. I am looking forward to seeing this work published.

**Reply:**

We sincerely thank the referee for their positive assessment and for the exceptional level of detail provided across both review rounds. The extraordinary care and rigor of their feedback made a substantial difference to the quality of this paper.

**Changes:**

No changes were made in response to this comment.

## R2 Response to Referee B

**Comment #1:**

After reading the response of the authors to the reviewer's comments, I recommend proceeding with publication of this revised version of the manuscript.

**Reply:**

We thank the referee for the positive evaluation of our revised manuscript and for the time and effort devoted to reviewing our work throughout the review process.

**Changes:**

No changes were made in response to this comment.

## R3 Additional Changes

Here, we summarize all minor changes made in the revised manuscript and Supplementary Materials that have not been addressed in the responses above:

1. We consistently replaced "source mixture model(s)" with "source-mixture model(s)" throughout the manuscript.
2. We consistently replaced "bin mode acquisition" with "bin-mode acquisition" throughout the manuscript.
3. We removed the abbreviation "NS" from the manuscript, as it was not used elsewhere.
4. To avoid redundant wording, the term "manufacturer-certified" was removed from the manuscript.
5. In accordance with the editor's formatting guidelines, we added DOI numbers to the data and code references in the reference list.
6. We removed the trailing commas following the subfigure labels in the caption of Fig. 1 to comply with the journal's formatting requirements.
7. We revised the vector notation for the experimental condition vector $\boldsymbol{\mathfrak{d}}$ throughout the manuscript to ensure that it is consistently displayed in bold in accordance with the journal's formatting guidelines.
8. We replaced the time symbol $t$ in Eq. 3 with the experimental condition vector $\boldsymbol{\mathfrak{d}}$ to ensure consistent terminology throughout the manuscript.
9. We ensured consistent use of the abbreviation "HPC" for high-performance computing throughout the manuscript after its first occurrence.
10. We ensured consistent use of the abbreviation "GNSS" for global navigation satellite system throughout the manuscript after its first occurrence.
11. In Section 4.3, we removed the statement "distributed across $10^2$ statistically independent simulation batches initialized with different random seeds for parallel execution and improved statistical sampling on the HPC cluster," as it did not provide relevant information for reproducibility.
12. We revised the Code Availability Statement to provide additional details on the software used for figure creation.
13. To comply with the editorial policies, all protected logos were removed from Fig. 1 and, where applicable, replaced with verbatim text labels.
14. To comply with the editorial policies, the email addresses of the co-authors were removed from the front page, retaining only the corresponding author's email address.
15. To comply with the editorial policies, the funding declaration was moved from the Acknowledgments section to a separate section entitled "Funding" at the end of the manuscript.
16. To comply with the editorial policies, image attribution statements were added to Figs. 2, 4–6.
17. To comply with the editorial policies, the cross-references in the legends of Figs. 5 and 6 were extended to explicitly reference panels a–d.

18. To comply with the editorial policies, the supplementary figures and tables in the Supplementary Information File were renamed to “Supplementary Fig. XX” and “Supplementary Table XX”, respectively. The corresponding cross-references were revised throughout both the main manuscript and the Supplementary Information File.

All modifications are fully traceable in the attached LaTeX `diff` marked-up manuscript.

## R4 Bibliography


1. Baldoncini, M. *et al.* Investigating the Potentialities of Monte Carlo Simulation for Assessing Soil Water Content via Proximal Gamma-Ray Spectroscopy. *J. Environ. Radioact.* **192,** 105–116. ISSN: 18791700. PMID: 29909224 (Dec. 1, 2018).
2. Kastlander, J. & Bargholtz, C. Efficient in Situ Method to Determine Radionuclide Concentration in Soil. *Nucl. Instrum. Methods Phys. Res. Sect. Accel. Spectrometers Detect. Assoc. Equip.* **547,** 400–410. ISSN: 0168-9002 (Aug. 1, 2005).
3. Namito, Y. *et al.* Transformation of a System Consisting of Plane Isotropic Source and Unit Sphere Detector into a System Consisting of Point Isotropic Source and Plane Detector in Monte Carlo Radiation Transport Calculation. *J. Nucl. Sci. Technol.* **49,** 167–172. ISSN: 0022-3131 (Feb. 2012).
4. Sellke, T., Bayarri, M. J. & Berger, J. O. Calibration of $\rho$ Values for Testing Precise Null Hypotheses. *Am. Stat.* **55,** 62–71. ISSN: 0003-1305 (Feb. 1, 2001).
5. Trotta, R. Bayes in the Sky: Bayesian Inference and Model Selection in Cosmology. *Contemp. Phys.* **49,** 71–104. ISSN: 00107514 (Mar. 2008).
